# Printing Microbial Dark Matter: Using Single Cell Dispensing and Genomics to Investigate the Patescibacteria/Candidate Phyla Radiation

**DOI:** 10.3389/fmicb.2021.635506

**Published:** 2021-06-16

**Authors:** Sandra Wiegand, Hang T. Dam, Julian Riba, John Vollmers, Anne-Kristin Kaster

**Affiliations:** ^1^Institute for Biological Interfaces 5, Karlsruhe Institute of Technology, Karlsruhe, Germany; ^2^Laboratory for MEMS Applications, IMTEK - Department of Microsystems Engineering, University of Freiburg, Freiburg, Germany; ^3^Institute for Applied Biosciences, Karlsruhe Institute of Technology, Karlsruhe, Germany

**Keywords:** single amplified genome (SAG), co-assembled genomes (CAGs), metagenomics, metagenome-assembled genomes (MAGs), bacteria

## Abstract

As of today, the majority of environmental microorganisms remain uncultured. They are therefore referred to as “microbial dark matter.” In the recent past, cultivation-independent methods like single-cell genomics (SCG) enabled the discovery of many previously unknown microorganisms, among them the Patescibacteria/Candidate Phyla Radiation (CPR). This approach was shown to be complementary to metagenomics, however, the development of additional and refined sorting techniques beyond the most commonly used fluorescence-activated cell sorting (FACS) is still desirable to enable additional downstream applications. Adding image information on the number and morphology of sorted cells would be beneficial, as would be minimizing cell stress caused by sorting conditions such as staining or pressure. Recently, a novel cell sorting technique has been developed, a microfluidic single-cell dispenser, which assesses the number and morphology of the cell in each droplet by automated light microscopic processing. Here, we report for the first time the successful application of the newly developed single-cell dispensing system for label-free isolation of individual bacteria from a complex sample retrieved from a wastewater treatment plant, demonstrating the potential of this technique for single cell genomics and other alternative downstream applications. Genome recovery success rated above 80% with this technique—out of 880 sorted cells 717 were successfully amplified. For 50.1% of these, analysis of the 16S rRNA gene was feasible and led to the sequencing of 50 sorted cells identified as Patescibacteria/CPR members. Subsequentially, 27 single amplified genomes (SAGs) of 15 novel and distinct Patescibacteria/CPR members, representing yet unseen species, genera and families could be captured and reconstructed. This phylogenetic distinctness of the recovered SAGs from available metagenome-assembled genomes (MAGs) is accompanied by the finding that these lineages—in whole or in part—have not been accessed by genome-resolved metagenomics of the same sample, thereby emphasizing the importance and opportunities of SCGs.

## Introduction

Prokaryotic microorganisms are the oldest, most abundant, and particularly most diverse forms of life on Earth and dominate many functions of the biosphere. However, it is estimated that most bacterial and archaeal clades from environmental microcosms remain uncultured (Hedlund et al., 2014; Lloyd et al., 2018; Steen et al., 2019). Over the past decade, major advancements have been made in the study of uncultured organisms (Nayfach et al., 2020). Together, these novel approaches have led to the discovery of 3,087 (November 2020, GOLD database) previously unknown genomes belonging to a monophyletic, genetically distinct group termed Candidate Phyla Radiation (CPR) (Wrighton et al., 2012; Brown et al., 2015; Anantharaman et al., 2016) or, Patescibacteria (Rinke et al., 2013; Parks et al., 2017, 2018). This taxon currently comprises about 73 phylum-level taxa (Castelle and Banfield, 2018), including the superphyla *Microgenomates* (candidate division OP11) and *Parcubacteria* (OD1) as well as diverse other phyla such as *Cand. Saccharibacteria* (TM7) and *Cand. Gracilibacteria* (BD1-5) (Brown et al., 2015; Hug et al., 2016; Danczak et al., 2017; Parks et al., 2020). Strains of the Patescibacteria/CPR most likely constitute a significant share of the bacterial domain, as they have been suggested to represent between 15 and 26.3% of the bacterial linages (Brown et al., 2015; Parks et al., 2017; Schulz et al., 2017). Many Patescibacteria/CPR genomes appear to lack ubiquitous biosynthetic pathways, e.g., for the production of amino acids, nucleotides, cofactors, and membrane lipids, while protein families linked to cell-cell interactions are frequently found within this group (Luef et al., 2015; Castelle and Banfield, 2018; Meheust et al., 2019). Patescibacteria/CPR also has small cell and genome sizes and seem to be widespread in highly diverse habitats in spite of their reduced genomes. They were therefore mostly suspected to be symbionts of other prokaryotic cells, a conclusion backed up by the observation of *Cand. Saccharibacteria* being epibionts of Actinobacteria (Castelle and Banfield, 2018; Cross et al., 2019; Geesink et al., 2020). However, a recent survey on putative cell-cell associations did not show an enrichment of Patescibacteria/CPR in these associations, thereby disputing the idea of the symbiotic nature of the clade (Beam et al., 2020). Since the Patescibacteria/CPR are still understudied, there is not yet sufficient data to clarify whether the distinct phylogenetic position of the Patescibacteria/CPR in the tree of life is an effect of rapid evolution by genome reduction or due to a very early separation from non-Patescibacteria/CPR (Hug et al., 2016; Castelle and Banfield, 2018; Parks et al., 2018; Meheust et al., 2019).

With the exception of some recent progress in the field of targeted isolation and cultivation where co-cultures of *Cand. Saccharibacteria* could be established from oral samples (Cross et al., 2019; Murugkar et al., 2020), most of the techniques that enabled these findings are independent of cultivation and instead rely on the recovery of genomes, either based on genome-resolved metagenomics—the shotgun sequencing of the complete biome of a sample and reconstruction of metagenome-assembled genomes (MAGs)—or single-cell genomics (SCG), where cells of community members are separated and sequenced individually (Tyson et al., 2004; Lasken, 2007; Blainey, 2013). 96% (November 2020, GOLD database) of the currently available Patescibacteria/CPR genomes are MAGs from shotgun sequencing of complete communities, with no pure isolates and only few genomes derived from co-cultures so far. MAGs in general, however, have some drawbacks, especially when it comes to the exact nature of individual constituents of the examined community—information on genetic heterogeneity of related organisms will be lost as the assembly usually collapses subtle differences and information on single cells cannot be resolved (Woyke et al., 2017).

Some of these issues can be addressed by focusing on individual cells and the reconstruction of single amplified genomes (SAGs) (Marcy et al., 2007; Podar et al., 2007). This would allow capturing laterally transferred “genomic island” regions within the genomes and also enable the analysis of members of the rare biosphere that would be missed during binning as well as cell-cell associations that are omitted in MAG construction (Woyke et al., 2017; Dam et al., 2020). Different methods have been developed to enable the first step of this process, the separation of individual cells (Kaster and Sobol, 2020). In brief, these methods can be differentiated into techniques that first identify cells of interest and then mechanically separate them from the sample (micromanipulation) or techniques that singularize the cells by random encapsulation and then analyze and sort each droplet (Blainey, 2013). Most SCG studies make use of use of the latter, predominantly in the form of fluorescence-activated cell sorting (FACS) (Stepanauskas and Sieracki, 2007; Woyke et al., 2017). The major advantage of this method is not only its high throughput, but also that it allows the use of different, fluorescently labeled probes to target specific cells (Hatzenpichler et al., 2016; Dam et al., 2020; Doud et al., 2020). Additionally, and in contrast to most other techniques, FACS systems are established technology that are commercially available (Blainey, 2013; Gross et al., 2013). However, the method comes with its own problems and obstacles, such potentially causing cell stress by staining as well as *via* shearing forces that can occur during sorting (Mollet et al., 2008; Blainey, 2013; Burke et al., 2013), possibly affecting cell and genome recovery as well as potential alternative downstream applications such as cultivation or transcriptomics. Furthermore, the fact that morphological information, such as cell size and form, is not visually recorded directly but only indirectly assessed *via* the intensity and duration of fluorescence signals, may make it difficult to target special cell types and morphologies (Blainey, 2013; Kodzius and Gojobori, 2016; Woyke et al., 2017; Kaster and Sobol, 2020).

To overcome some of these issues, we here describe the use of an alternative technology for label-free single-cell isolation of prokaryotes, a single-cell printer (Gross et al., 2013). Previous studies demonstrated that the instrument is capable of efficiently isolating single cells from both eukaryotic and bacterial samples, such as *E. coli* cultures (Gross et al., 2013; Stumpf et al., 2015; Riba et al.,2016a,b), making this an interesting tool for the cultivation of understudied bacterial clades from environmental samples. The beginnings of cell printing were made by repurposing inkjet print heads, as they were able to generate droplets only slightly larger than eukaryotic cells (Wilson and Boland, 2003). The method was refined to enable the encapsulation of single cells in picoliter sized droplets that can be deposited on various surfaces upon visual inspection (Saunders et al., 2008; Yusof et al., 2011). The single-cell printer has a transparent dispensing microchip for on-demand drop generation that is self-filling by capillary forces. The nozzle of the chip is continuously monitored by a video microscope. Before a droplet is formed, the number and morphology of the cells in the nozzle is determined using automated image processing. The chip has a silicon membrane back that is then deflected by a piezo-electronic piston to generate the droplet, which is subsequentially either removed by a vacuum shutter or subjected to further analysis in a microwell plate (Gross et al., 2013; Riba et al., 2016a).

Here, we utilize the microfluidic single-cell dispenser system for the first time to isolate microorganisms from a complex environmental sample. The sample source was a wastewater treatment plant that has previously been shown to be rich in Patescibacteria/CPR bacteria (Dam et al., 2020). Through this study we were able to increase the knowledge on the genomic diversity of this understudied clade. We could demonstrate that some of the major drawbacks of FACS, e.g., the lack of single-cell visualization (and therefore proof of consistent cell separation) and suspected pressure induced cell stress (Mollet et al., 2008; Binek et al., 2019) that may affect cell yield and purity during FACS sorting can be positively addressed, enabling a variety of alternative downstream applications for single cell sorting.

## Materials and Methods

### Sample Collection and Processing

The sampling and processing was previously described by Dam et al. (2020). In brief, a wastewater sample from the aerated lagoon (LEA) of the wastewater treatment plant of the Establecimiento Juanicó winery (located in the village Juanicó in Canelones, Uruguay, latitude −34.6, longitude −56.25) were collected 20 cm below the water level. The sample was vortexed at maximum speed for 3 min to release cells attracted loosely to the sediments. After 1 h, the sample was centrifuged at 2,500 rpm for 30 s to remove large particles (Rinke et al., 2014). The supernatant was filtered through a 30 μm polycarbonate membrane using gravity flow filtration (Celltrics^®^ Filter, 508 Partec, Münster, Germany).

### DNA Extraction for Metagenome Sequencing

DNA was extracted from the samples using a hexadecyltrimethylammonium bromide (CTAB)-based method with some modifications (Griffiths et al., 2004) as previously described by Dam et al. (2020). 1.5 mL of the filtered samples was centrifuged at 21,000 × *g* for 5 min to collect biomass. Pellets were then transferred into a Lysing matrix E tube (MP Biomedicals, France). 500 μL 6% CTAB extraction buffer and 500 μL phenol:chloroform:isoamyl alcohol (25:24:1) were added to the extraction tube. Cells were lysed by vortexing at 21,000 × *g* on a Vortex Genie2 (Scientific Industries, NY, United States) for 3 min. Supernatant was extracted twice with phenol:chloroform:isoamyl alcohol (25:24:1) and twice with chloroform:isoamyl alcohol (24:1). The aqueous phase was transferred into a clean 1.5 mL tube. DNA was precipitated with 2.5 volume of 100% ethanol and 0.1 volume of 3 M sodium acetate (pH 5.2) and re-suspended in 50 μL PCR grade water. Extracted DNA was cleaned up with the DNA Clean and Concentrator-5 kit (Zymo Research, Germany) as per the manufacturer’s instruction.

### Untargeted Single-Cell Dispensing

Shortly before single-cell dispensing with a prototype single-cell printer that was modified for dispensing bacteria (Riba et al., 2016a), the filtered wastewater sample was diluted 1:100 with sterile filtered PBS and passed through a membrane filter with 10 μm pore size (Celltrics^®^ Filter, 508 Partec, Germany). However, no frozen glycerol stocks from this exact timepoint, are available anymore for additional sorts. 30 μL of the sample was then pipetted into a dispensing cartridge with 20 μm nozzle size (cytena GmbH, Germany). Prior to dispensing, an ionized air blower (minION2, SIMCO-ION, Netherlands) was directed onto the microwell plates for 30 s to remove electrostatic charges, which could cause deflection of the free flying droplets. 880 single cells (Supplementary Figure 1) were dispensed into five half standard 384-well PCR plates (with 16 empty wells as negative controls per plate) using the single-cell printer for bacteria as described previously (Riba et al., 2016a). To ensure that the droplets with single cells were deposited precisely into the center of each well, the system for automated dispenser offset correction was used, as described previously (Riba et al., 2016b). After single-cell dispensing the plates were sealed with adhesive aluminum PCR plate foils and stored at −80°C.

### Multiple Displacement Amplification

Cells were lysed in 0.7 μL lysis buffer and their genomic DNA was released during alkaline lysis at 65°C for 10 min. Genomic DNA was amplified with phi29 DNA polymerase at 30°C for 6 h using REPLI-g^®^ Single Cell Kit (Qiagen, Hilden, Germany) on a CFX384 Touch^TM^ Real-Time Detection System (Bio-Rad Laboratories, Munich, Germany) in 5 μL reaction volume. WGA was monitored in real time by detection of SYTO13^®^ (Life Technologies, CA, United States) fluorescence every 5 min. Multiple displacement amplification (MDA) reaction was then terminated at 65°C for 10 min. The cycle quantification (Cq) values and endpoint relative fluorescence units were used to determine positive WGA reactions. *E. coli* served as positive control for this reaction.

### 16S rRNA Gene PCR-Based Screening

Multiple displacement amplification products were diluted 1:20 and used as templates to amplify 16S rRNA genes with the universal bacterial primer pair 926wF (5′-AAACTYAAAKGAATTGRCGG-3′) and 1392R (5′-ACGGGCGGTGTGTRC-3′) (Rinke et al., 2014). PCR products were cleaned with DNA Clean and Concentrator-5 (Zymo Research, Freiburg, Germany) and subjected to Sanger sequencing. The resulting reads were trimmed and filtered by Trimmomatic v.0.36 (Bolger et al., 2014) with the following parameters: HEADCROP:80 LEADING:50 TRAILING:30 SLIDINGWINDOW:40:36 MINLEN:150. Remaining sequences submitted to the web- based SINA Aligner and SINA Search and Classify for preliminary classification (Pruesse et al., 2012).

### Library Preparation for Shotgun Metagenomic and Single Cell Genome Sequencing

Metagenomic DNA extracts as well as sorted single cell MDA products were quantified using the Qubit dsDNA HS Assay Kit (ThermoFisher Scientific, OR, United States). Illumina Sequencing libraries were then prepared using the NEBNext^®^ Ultra^TM^ DNA Library Prep Kit and NEBNext^®^ UltraTM II FS DNA Library Prep Kit (New England BioLabs, Frankfurt, Germany), respectively, using unique dual index adapters and following the manufacturer’s instruction. Resulting library fragment lengths were assessed using the Agilent 2100 Bioanalyzer with a High Sensitivity DNA Kit (Agilent Technologies, Germany). The libraries were then pooled and sequenced on an Illumina NovaSeq system using a paired-end approach with 150 cycles per read. Sequencing depths of approximately 6.8 million read pairs or 2 Gb were produced per SAG, on average.

### Read Processing, Assembly and Binning

Read processing consisted of a preliminary quality trimming and adapter clipping step using Trimmomatic v.0.36 (Bolger et al., 2014), bbduk v.35.69 (Bushnell, 2014) and cutadapt v.1.14 (Martin, 2011) in direct succession, followed by a merging step for combining overlapping read pairs into longer single reads using FLASH v.1.2.11 (Magoc and Salzberg, 2011) and a final filtering step for removing potential residual PhiX contamination using fastq_screen v.0.4.4 (Wingett and Andrews, 2018). For all steps, the respective program settings were as previously described in Dam et al. (2020). Metagenomic as well as single cell assemblies were done using SPAdes v.3.10.1 (Nurk et al., 2013) with k-mer lengths ranging from 21 to 101 in steps of 10. For assembly of SAGs as well as combined cell amplified genomes (CAGs) the “–careful” and “–sc” flags were applied, while the “–meta” flag was used for metagenome assemblies. Binning results obtained from the metagenome in parallel *via* Maxbin v.2.2.6 (Wu et al., 2015), and MetaBat v.2.12.1 (Kang et al., 2015) were integrated using DAS Tool v.1.1.1 (Sieber et al., 2018). Potential contaminant contigs and scaffolds were filtered from each bin based on the same approach described below in chapter “Assembly assessment and taxonomic classification.”

### Assembly Assessment and Taxonomic Classification

For the metagenome, 16S ribosomal RNA genes was predicted using RNAmmer v1.2 (Lagesen et al., 2007) and classified using SINA in conjunction with the SILVA database (Pruesse et al., 2012; Yilmaz et al., 2014). Protein coding genes were identified using prodigal v.2.6.3 (Hyatt et al., 2010). Universal single copy protein coding marker genes were then extracted using fetchMG v.1.0^1^, aligned against the ncbi nr database and classified based on the LCA approach implemented in kronatools (Ondov et al., 2011). Contig coverages were determined *via* read mapping using BamM v.1.7.3^2^. Relative taxon abundances in metagenomic samples were determined by associating the taxonomic classifications of each universal marker gene (protein coding as well as rRNA) independently with the corresponding contig coverages. For SAGs and CAGs, automatic functional annotations were performed using prokka v.1.14.5 (Seemann, 2014). A preliminary taxonomic evaluation was performed using a hierarchical contig classification approach (HCC), based on the “highest ranking” phylogenetic marker found on each contig (in decreasing order: rRNA genes, single copy house-keeping genes or total proteins) as previously described (Pratscher et al., 2018; Dam et al., 2020). Based on these classifications, the most dominant taxon assignments were determined for each SAG and MAG, and any contig with clearly contradicting LCA classifications supported by average BLAST-identities above 40% was marked as potential contamination, and subsequentially filtered from the assemblies before submitting to NCBI, also as previously described (Dam et al., 2020). This is a very strict filtering process, designed as a precautionary measure to minimize the influence of potential artifacts on public genome databases, caused by trace contaminations known to occur during single cell genome sequencing due to residual DNA fragments in the MDA reagents (Woyke et al., 2011) or even due to free environmental DNA fragments co-sorted with single cells (Rodrigue et al., 2009). Such artifact contigs may easily missed by conventional screening processes (such as CheckM) if no conserved marker genes are encoded. As a result, about 10% of the contigs in each assembly were filtered out from the final assemblies on average. Genome quality assessments were done with CheckM (Parks et al., 2015). Contamination was estimated based on marker gene duplications according to the MISAG standard, but paralog-corrected by subtracting the fraction of near identical marker-gene copies [CheckM’s “strain heterogeneity” (SH)], which are most unlikely to represent actual cross-species contamination, from the total fraction of duplicate marker genes [CheckM’s “contamination” (C)] according to the formula: C − [(SH/100) × C]. “Predicted genome size” was determined by relating the size of the SAG to its “completeness.” The average nucleotide identity (ANI) was calculated using OrthoANI (Lee et al., 2016). The SAGs and CAGs were submitted to NCBI WGS under BioProject number PRJNA664701.

### Phylogenetic Inference

To infer the taxonomy of the SAGs, the full-length 16S rRNA gene was re-classified by SINA and the SILVA database (Pruesse et al., 2012; Yilmaz et al., 2014). Afterward, the aligned sequences were joined with the SILVA database (version 138 SSU, December 2019), sequences identities were determined using the Arb software package (Westram et al., 2011) and phylogenetic trees were constructed (Supplementary Figure 2). Additionally, the SAGs were classified on marker gene-level by GTDB-Tk with GTDB r89 (Parks et al., 2018, 2020). For further genome-based analyses, related genomes were downloaded from GenBank if *N*_50_ > 35 kb and were only used when completeness was >70% and paralog-corrected contamination was <5%. All genomes were re-annotated with prokka v.1.14.5 (Seemann, 2014) to ensure comparability. GTDB-Tk analysis was redone and the resulting alignment was used for the calculation of the marker gene-based protein alignment tree. The novelty of the SAGs and CAGs was determined by employing PhyloRank v0.0.37^3^ on the same tree and the taxonomy file gained from GTDB-Tk. Orthologous genes shared between SAGs, CAGs and selected reference genomes were determined *via* the bidirectional BLAST approach implemented in Proteinortho6 (Lechner et al., 2011), and used for additional phylogenetic clustering based on the “Multi Locus Sequence Analysis” (MLSA) approach using custom python scripts as previously described in Howat et al. (2018) and Wiegand et al. (2020) (Supplementary Figures 3–6). All phylogenetic trees were constructed using FastTree (JTT model, 1,000 resamplings) (Price et al., 2010) and visualized with iTOL v4 (Letunic and Bork, 2019). ANIs were calculated using OrthoANI (Lee et al., 2016) and average amino acid identities (AAI) were gained with the aai.rb script of the enveomics collection (Rodriguez-R and Konstantinidis, 2016).

CAGs were obtained by merging the respective datasets of SAGs that displayed 16S rRNA gene identities of 100% and/or ANI values >98.4%.

### Genome Analysis

The metabolic analyses of the SAGs have been done with eggNOG 5.0 (Huerta-Cepas et al., 2019), BlastKoala (Kanehisa et al., 2016) and KEGG, as well as InterProScan v5.44-79 (Mitchell et al., 2019). For the analysis of horizontal gene transfer, genome similarity has been determined by ANI, matching regions were determined by blastn and the gene content was evaluated by using prokka v.1.14.5 (Seemann, 2014) followed by Proteinortho6 analysis (Lechner et al., 2011).

## Results and Discussion

### Patescibacteria/CPR in a Winery Wastewater Treatment Plant

In a recent study (Dam et al., 2020), we analyzed the microbial community of the on-site wastewater treatment plant of the Juanicó winery in southern Uruguay. More particularly, sludge from the aerated lagoon (Laguna de Ecualizacion y Aireacion - LEA) of the plant was analyzed over the course of 3 years (2013–2015). While this study focused mainly on members of the *Chloroflexi* phylum found in the specimens, some other noteworthy results were found: In the metagenomic analysis of the 2015 sample, 39% of the total 16S rRNA gene sequences and 20% of all contigs defined by protein marker genes belonged to the Patescibacteria/CPR. The latter value was also supported by the finding, that 17% of all MAGs were classified as members of the Patescibacteria/CPR (Dam et al., 2020).

In this proof-of-principle study, we wanted to examine the feasibility to use the above described single-cell printer to sort unlabeled prokaryotic cells from an environmental sample with high representation of Patescibacteria/CPR community members. In a first step, shotgun metagenomic sequencing data of the sample were re-analyzed to confirm the high abundance of Patescibacteria/CPR.

**FIGURE 1:**
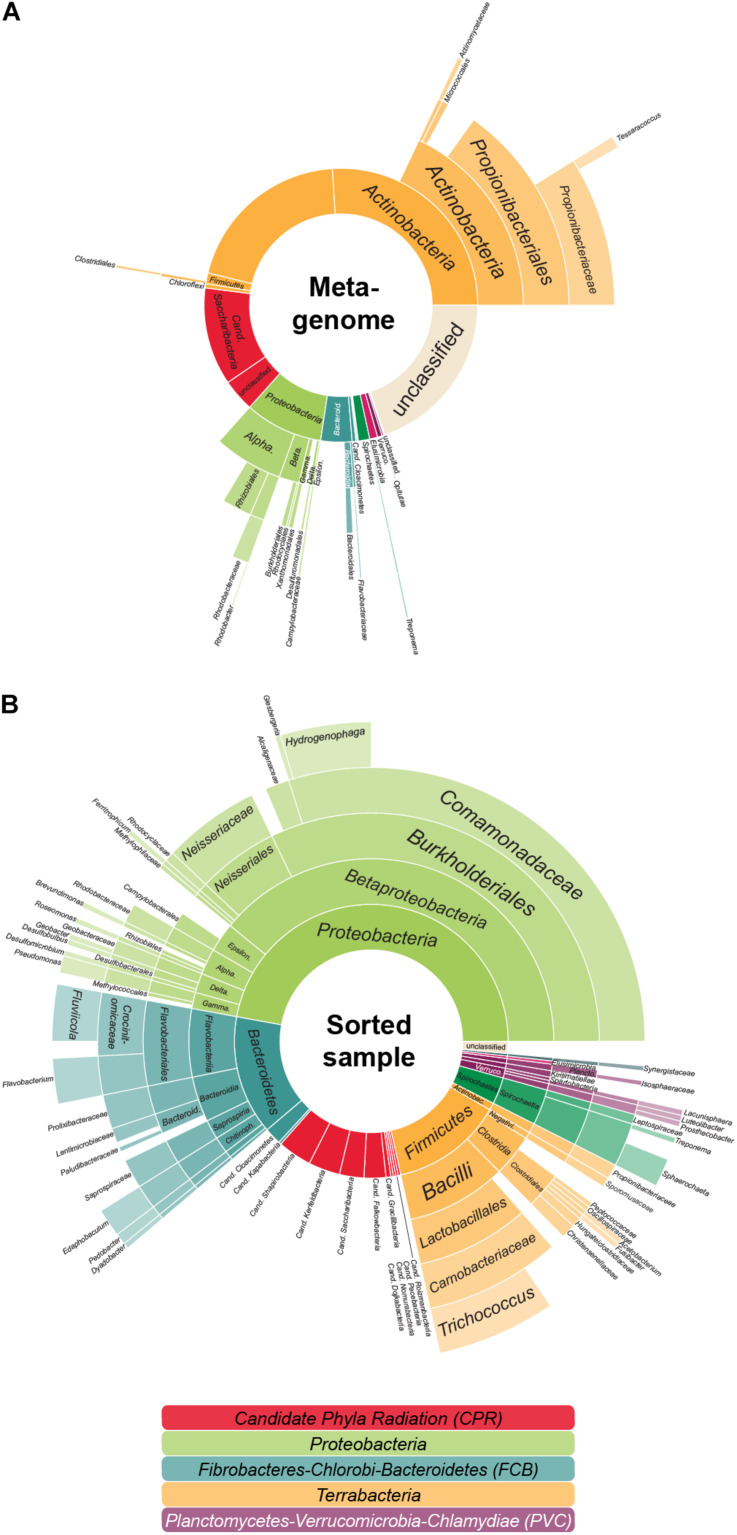
The aerated lagoon (LEA) of a winery wastewater treatment plant—before and after sorting. **(A)** Microbial community composition. In total, 26.4 GB of shotgun metagenomic sequencing data of the sample were processed, assembled and analyzed based on the phylogenetic classification of all attributable marker genes. In total, 291,396 marker genes could be identified. **(B)** Taxonomic composition of cells after cell sorting. Relative proportion of the covered taxa by 16S rRNA gene sequencing inferred for those sorted cells accessible to cell lysis, whole genome amplification and PCR. In total, 359 evaluable Sanger sequencing reads were gained from 880 sorted cells. The colors reflect the higher-level unranked taxa/superphyla encompassing the different phyla.

In the previous analyses by Dam et al. (2020), single copy marker protein sequences were used to infer taxonomic information of the metagenome: contigs were classified based on the most relevant marker and taxa were then quantified by the coverage of each contig. In this approach, each contig is considered equally—no matter the number of encoded marker genes. Thereby larger contigs with several marker genes might be underestimated when compared to several small contigs with one marker gene each. In order to ensure that highly fragmented genomes are not favored over genomes that assemble more easily—such as the relatively small Patescibacteria/CPR—we here use a modified approach that quantifies each taxon by the coverage of all universal single copy marker genes (that should be present in each genome no matter the size), not the encoding contigs. Interestingly, with this method (Figure 1A and Supplementary Table 1), the estimated amount of Patescibacteria/CPR members in the LEA sample decreased slightly in comparison to the above mentioned 20% (Dam et al., 2020). This finding implies that the Patescibacteria/CPR in this metagenome sample are relatively fragmented despite their high abundance, indicating that they may represent a heterogenous group consisting of closely related but diverse, low abundant species. Nonetheless, with a total abundance of 15.4%, Patescibacteria/CPR as a group is confirmed to represent a significant constituent of the community.

### Single-Cell Printing of an Environmental Sample

**FIGURE 2:**
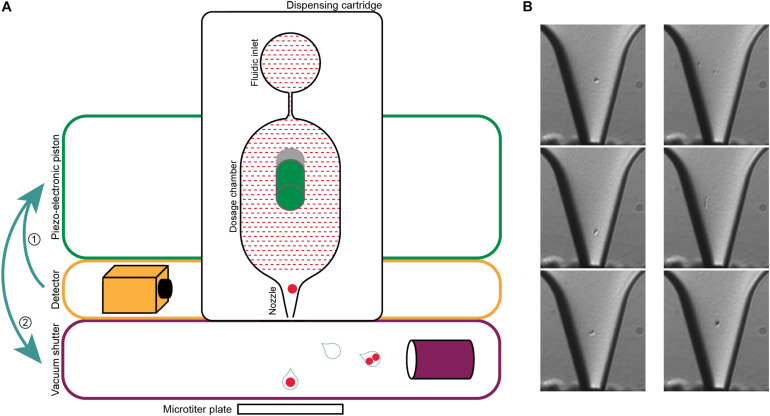
Single-cell printer and single cell micrographs. **(A)** Schematics of the single-cell printer. Upon detection of cells in the nozzle of the dispensing cartridge, the piezo-electronic piston deflects the back of the dosing chamber. The displacement of the liquid suspension inside the chip (red shaded area) enforces the release of a droplet that is then either captured in a microtiter plate (single cell) or removed by vacuum suction (all other cases). Adapted from Gross et al. (2013). **(B)** Micrographs of single cells (left panel) and two cells (right panel) in the nozzle. The images are recorded at the time of detection, thereby allowing the linkage of every micrograph to the corresponding micro-well. The occurrence of two cells in one drop might either be two independent cells (top), a cell in a late stage of division (middle) or two attached cells that might be of different origin (bottom). Scale bar is 10 μm.

In this study, we demonstrate that the previously described single-cell printer (Riba et al., 2016a) allows for label-free isolation of individual bacteria from complex microbial samples (Figure 2). The system is based on a drop-on-demand dispenser, a bright-field video microscope, and automated image processing to encapsulate single bacteria in free flying 35 pL droplets. Here, we used the system to isolate 880 cells of the LEA sample. Each cell was subjected to multidisplacement amplifications (MDAs), of which a total of 717 (81.5%) were successful based on real-time amplification data (Supplementary Figure 1). Usually, MDA success rates from FACS protocols, where the cells are either treated with fluorescent stains or labeled with fluorescence *in situ* hybridization (FISH) probes, are variable but tend to be under 40% (Clingenpeel et al., 2014; Kaster et al., 2014; Rinke et al., 2014; Dam et al., 2020). The extraordinarily high amplification success rate derived from sorting with the single-cell printer indicates a relatively high yield of amplifiable cellular DNA. Reasons for this might be the relatively gentle and precise deposition of cells due to absence of high pressures that could lead to burst and emptied cell envelopes, as well as the lack of fixation reagents that could affect the integrity of the cell as well as the DNA (Kodzius and Gojobori, 2016; Woyke et al., 2017; Kaster and Sobol, 2020). The question to which degree FACS sorting, on the other hand, may impact the integrity of sorted cells is still surprisingly inconclusive. Significant mechanical cell damage has been described in eukaryotic cell lines and attributed to the high pressure and resulting hydrodynamic forces applied during FACS (Mollet et al., 2008; Binek et al., 2019). However, a recent analysis of this so-called “sorting induced cell stress” (SICS), concluded that the impact of shearing forces during FACS was negligible, at least for the viability of human blood cells (Pfister et al., 2020), while reports of stress induced changes in cell properties such as gene expression and metabolome appear to vary drastically between studies (Richardson et al., 2015; Llufrio et al., 2018; Binek et al., 2019).

An influence of shearing forces has also been suspected for FACS sorting of bacterial cells (Blainey, 2013), and experiments with morphological *E. coli* mutants revealed drastically lower viability rates for filamentous compared to non-filamentous cells (Burke et al., 2013), indicating that different cell types may be differently affected. However, depending on the method or stain applied, viability may be affected by staining as much as by shearing.

DNA extractions from bulk-sorted (>5 million) cells from *E. coli* pure cultures as well as soil samples performed *via* FACS show that the relative proportion of free DNA to cell-associated DNA increased drastically after FACS sorting compared to unsorted samples (Supplementary Table 2), indicating possible cell damage and subsequent release of DNA into the surrounding medium/buffer. The soil samples were apparently far more affected than pure *E. coli* cultures, likely due to the presence of dead/damaged cells as well as free environmental DNA. While this would mostly seem to affect other possible downstream applications than SCG, such as cultivation or single cell transcriptomics, it may well also affect the genomes that can be captured if extensive cell lysis occurs before or during droplet formation (premature cell lysis). It appears reasonable that the high MDA success rate (>80%) observed after sorting with the single cell printer may be linked to its specific sorting properties that may cause lower cell stress and therefore less premature cell lysis. Therefore samples with clumped or particle associated cells may have to be pre-treated, e.g., by sonification, in order to ensure that cells are separated enough to be recognized as individual cells and sorted. This gentler handling of the sorted cells comes at the price of comparatively reduced throughput. Whereas the FACs can bulk sort millions of cells into the same well within hours, the same process may take days with the single cell printer. However, since such extremely large-scale bulk sorts are hardly standard procedures for single cell genomics, the difference in throughput has no negative effect for standard applications of sorting individual cells into 384 well plates. With the prototype single-cell dispensing system used in this study, 384 single-cell were dispensed in ∼25 min. Considering the short time for setting up the instrument (∼5 min) and that there are no cleaning procedures required due to disposable cartridges, the overall time required for single-cell isolation is significantly shorter than typically experienced with FACS sorting. In the meantime, commercial versions of the single-cell dispensing system (b.sight, cytena GmbH, Germany) have been developed which allow even faster processing due to increased dispensing frequency.

Of the 717 successfully MDAed cells, 420 (58.6%) yielded positive 16S rRNA gene PCR products and of those, 359 (50.1%) resulted in interpretable Sanger sequencing reads (Supplementary Figure 1 and Supplementary Table 3). One half of the WGA not being interpretable by 16S rRNA gene PCR might be a multifactorial problem: PCR primers may not bind universally enough to target the 16S rRNA gene, as has been shown in the past (Takahashi et al., 2014; Eloe-Fadrosh et al., 2016), insufficient 16S rRNA gene templates due to an incomplete MDA reaction might prevent a successful PCR, or the presence of several 16S rRNA genes that could be introduced by either free DNA in the sample or the sorting of more than one cell would hamper Sanger sequencing.

Anyhow, based on 16S rRNA gene sequencing, about 14.5% of the sorted cells in the sample were Patescibacteria/CPR (Figure 1B), a result closely matching the 15.4% abundance resolved by shotgun metagenomic sequencing of the sample (Figure 1A). Based on these sequences, 50 MDA products representing the Patescibacteria/CPR were selected and subjected to short-read sequencing, read processing, and assembly. The assembled data were screened and filtered for potential contaminant contigs and scaffolds, possibly introduced by free DNA in the sample, or residual contaminations in reagents and on instruments. However, it can be assumed, that these contamination sources are less of an issue for single-cell printer sorts compared to FACS, as the lower pressure during sorting should reduce premature cell lysis, and the lower required buffer volumes represent less of a contamination hazard. For maximum sensitivity toward possible contaminants, a hierarchical contig classification approach (HCC) was employed (Pratscher et al., 2018; Dam et al., 2020). This method enables the detection of potential contaminant scaffolds even in the absence of highly conserved marker genes, which may be missed by commonly used genome assessment tools. Based on this rather strict approach, three samples were excluded from further analyses due to suboptimal MDA products and resulting low genome completeness while 14 WGAs seemed to contain contigs of two different species and for six WGAs the possible presence of three or more taxa was indicated (Supplementary Figure 1). The evaluation of the micrographs taken during the sorting process gave some insights into the reasons for these findings: some micrographs show multiple cells being spuriously sorted into one well or suggest the presence of cells associated to other microbial cells. Also, there are several potential sources for contaminants that might either be derived from free DNA in the sample or in the used WGA reagents (Rodrigue et al., 2009; Woyke et al., 2011). These issues may either be overcome by enhancing the employed detection algorithm or the used reagents and separation protocols. Another way might be to take advantage of the possibility to detect cell-cell interactions and specifically target these cells. However, eight of the 14 samples containing contigs of two different species seem to be derived from only one cell and for five of these the presence of contigs that distinctly belonged to two different taxa could be shown, e.g., *Cand. Falkowbacteria* being associated with *Cand. Cloacimonetes* and *Cand. Shapirobacteria* being associated with *Marinilabiliaceae*. While these occurrences might be due to the symbiotic nature of these organisms (Cross et al., 2019), 27 SAGs were confidently interpreted to be derived from one cell each (Table 1) and might therefore represent free-living members of the Patescibacteria/CPR clade (Beam et al., 2020).

**TABLE 1:** Patescibacteria/CPR SAGs and CAGs generated from successful sorting WGA.

		Estimated completeness	Contamination		MIMAG/MISAG standard	SAG/CAG size (Mb)	Predicted genome size (Mb)	GC	Taxonomy	Taxonomic evidence	Taxonomic novelty
			CheckM	Paralog-corrected							
**CAG1**		**68.8**	**0**	**0**	**mq**	**0.72**	**1.05**	**44.7**	***Cand. Saccharibacteria***	**sgm**	**Novel species**
	S2_E04	35.9	0	0	Lq	0.36	1	44			
	S3_L12	47	0	0	Lq	0.47	0.99	46.7			
**S5_F09**		**10.3**	**0**	**0**	**Lq**	**0.25**	**2.39**	**45.8**	***Cand. Saccharibacteria***	**sm**	**n. d.**
**CAG2**		**89.7**	**1.9**	**0.9**	**mq**	**1.33**	**1.5**	**32.1**	***Cand. Komeilibacteria***	**sgm**	**Same novel family as CAG3 but different genus**
	S1_H12	65.5	0.2	0.2	mq	0.77	1.17	31.5			
	S5_K02	61.9	0	0	mq	0.91	1.48	31.7			
	S5_K13	82.7	0	0	mq	1.06	1.28	31.5			
**CAG3**		**70.7**	**13**	**7.8**	**mq**	**1.34**	**2.13**	**33.3**	***Cand. Komeilibacteria***	**sgm**	**Same novel family as CAG2 but different genus**
	S1_I04	38.1	1.7	1.7	lq	0.63	1.72	32.8			
	S2_K09	55.7	4.7	4.7	mq	0.81	1.59	34			
	S3_J02	23.8	2.2	0.8	lq	0.55	2.37	30.9			
**S3_E16**		**12.1**	**4.3**	**0**	**lq**	**0.33**	**2.74**	**30.5**	***Cand. Komeilibacteria***	**sm**	**n. d.**
**CAG4**		**82.8**	**12.1**	**9.4**	**mq**	**2.17**	**2.96**	**44.4**	***Cand. Falkowbacteria***	**sgm**	**Novel genus**
	S1_L12	52.1	1.2	1.2	mq	0.63	1.23	40			
	S2_E10	40.5	4.7	3.5	lq	1.25	3.37	48.1			
	S2_J07	20.4	0.9	0	lq	0.34	1.66	41.5			
	S3_K08	50	1.7	1.7	mq	0.83	1.72	38			
	S3_L10	44.1	7.8	2.6	lq	0.42	1.02	38.8			
	S4_F15	36.7	0	0.9	lq	0.5	1.41	39.1			
	S5_F19	44	0	0	lq	0.61	1.39	37.7			
	S5_I03	71.2	2.6	1.3	mq	1.11	1.59	38.6			
**S1_J13**		**36.1**	**0**	**0**	**lq**	**0.42**	**1.18**	**35.1**	***Cand. Shapirobacteria***	**sgm**	**Novel species**
**S1_L17**		**29.8**	**0**	**0**	**lq**	**0.5**	**1.68**	**36.1**	***Cand. Shapirobacteria***	**sgm**	**Novel genus**
**S1_L14**		**39.9**	**1.7**	**1.7**	**lq**	**0.65**	**1.7**	**30.9**	***Cand. Shapirobacteria***	**sgm**	**Novel species, same genus as S2_F12, S3_E17, S4_G10**
**S2_F12**		**66.6**	**0**	**0**	**mq**	**0.86**	**1.3**	**34.7**	***Cand. Shapirobacteria***	**sgm**	**Novel species, same genus as S1_L14, S3_E17, S4_G10**
**S3_E17**		**23**	**0**	**0**	**lq**	**0.69**	**3**	**32.3**	***Cand. Shapirobacteria***	**gm**	**Novel species, same genus as S1_L14, S2_F12, S4_G10**
**S4_G10**		**23.4**	**0**	**0**	**lq**	**0.55**	**2.35**	**38.7**	***Cand. Shapirobacteria***	**sgm**	**Novel species, same genus as S1_L14, S2_F12, S3_E17**
**S4_G22**		**33**	**0**	**0**	**Lq**	**0.74**	**2.25**	**36.7**	***Cand. Roizmanbacteria***	**sgm**	**Novel species**
**S2_J21**		**50.7**	**0**	**0**	**mq**	**0.63**	**1.25**	**36.2**	***Cand. Dojkabacteria***	**sg**	**Novel genus**
**S5_H10**		**62.3**	**0**	**0**	**mq**	**0.95**	**1.53**	**36.2**	***Cand. Gracilibacteria***	**sgm**	**Novel family**

*n. d., not determined due to insufficient completeness. The names of SAGs start with a capital “S,” while CAGs are numbered. Indented, non-bold SAGs belong to the preceding CAG. Genomes were determined to be either medium (mq) or low quality (lq) according to MIMAG/MISAG standards (Bowers et al., 2017). The predicted genome size gives the putative size of a complete genome based on SAG/CAG completeness and size. The taxonomy was determined by analysis of the 16S rRNA gene (s, SILVA 138 SSU), a marker gene-based classification (g, GTDB r89) and MLSA based on shared protein genes (m). The different results were translated to NCBI taxonomy.*

**FIGURE 3:**
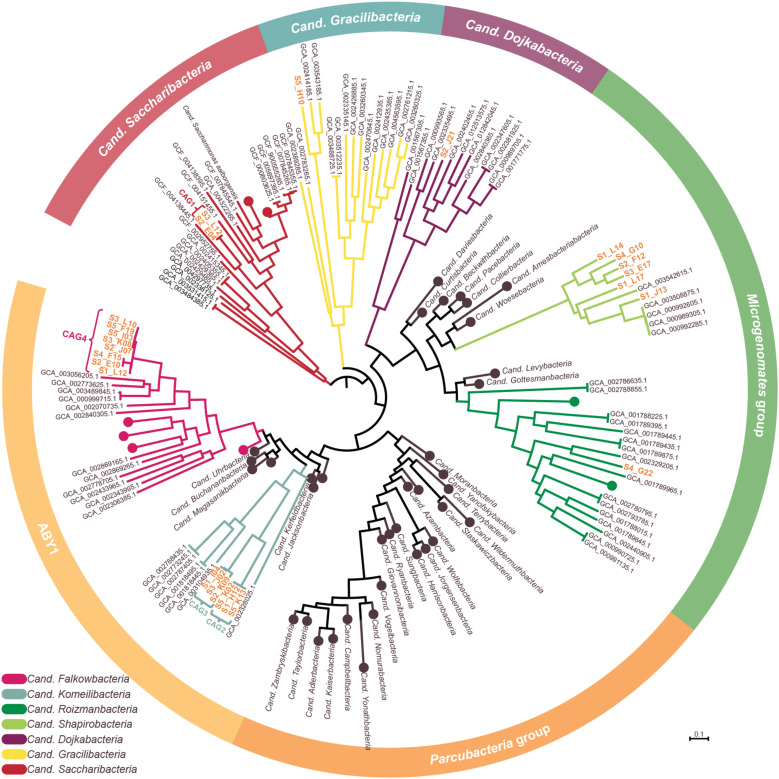
Phylogenetic representation of SAGs and CAGs of the Patescibacteria/CPR. The tree was calculated from a marker gene-based protein alignment (Chaumeil et al., 2019) with all accessible Patescibacteria/CPR genomes from GenBank above a quality threshold of scaffold *N*_50_ > 35 kb, completeness > 70% and paralog-corrected contamination < 5%. In total, the tree contains 975 Patescibacteria/CPR genomes and 71 genomes of the not shown Chloroflexi serving as outgroup. Clades with five or less branches were deleted from the tree for better readability and larger clades were collapsed and/or labeled based on the genomes assigned NCBI taxonomy. Two SAGs are not shown in the tree as they were too incomplete for GTDB classification. SAGs from this study are given in orange.

All gained SAGs range between 0.25 and 1.25 Mb in size, constituted by 57–1,269 scaffolds, and show paralog-corrected contamination ratios between 0 and 4.7%. Completeness values range from 10.3 to 82.7%, defining 10 SAGs as “medium-quality” and 17 as “low-quality” according to the MIMAG/MISAG standard (Alteio et al., 2020). The average completeness values of SAGs are usually highly variable throughout a sample (Hugoson et al., 2020) and generally lower than MAGs due to the biased nature of the MDA (Dichosa et al., 2012). With an average estimated completeness of 42.8% and average estimated contamination of 0.8%, our data are well within the quality range expected (Alneberg et al., 2018; Chijiiwa et al., 2020). Preliminary phylogenetic analyses indicated that some of the SAGs represent the same strains and could therefore be co-assembled to gain more complete genomes. For that, the requirements were 16S rRNA gene identities of 100% and/or ANI values > 95% (Supplementary Table 4). The validity of such groupings was then additionally verified *via* marker-gene based phylogeny (see below and Figure 3). On this basis, 16 SAGs were grouped and re-assembled into four so called co-assembled genomes (CAGs) (Table 1). These CAGs are more complete (68.8–89.7%) and larger in size (0.72–2.17 Mb) than the best of the respective comprising SAGs. Paralog-corrected contamination values were slightly increased but still below 10%. In summary, the 27 gained SAGs seem to belong to 15 different taxa represented by four CAGs and 11 SAGs.

### Phylogeny and Predicted Metabolism of Single Amplified Genomes From the Patescibacteria/CPR Lineage

To confidently infer the taxonomy of the genomes, multiple methods were employed. First, the corresponding full-length 16S rRNA genes were classified by SILVA (Supplementary Figure 2; Yilmaz et al., 2014). Additionally, SAGs were classified on marker gene-level by GTDB classification (Chaumeil et al., 2019; Parks et al., 2020). In a final step, phylogenetic relationships between all CAGs, SAGs and selected references were determined *via* multilocus sequence analysis (MLSA) based on ubiquitous single copy protein-coding genes (Supplementary Figures 3–6). As summarized in Table 1, the classifications derived from all three analyses were in total agreement in 11 out of 15 cases, while the completeness of four other SAGs was only sufficient to allow the application of two analytical methods which also were always in agreement. These results furthermore confirm that all SAGs that were selected for co-assembly form coherent clusters with each other as well as the corresponding resulting CAG, thereby supporting the validity of the CAGs. All 15 taxa were found to cluster within the Patescibacteria/CPR clade. They represent 15 different novel species belonging to *Cand. Shapirobacteria* (6 species), *Cand. Komeilibacteria* (3), *Cand. Saccharibacteria* (2), *Cand. Roizmanbacteria* (1), *Cand. Falkowbacteria* (1), *Cand. Gracilibacteria* (1), and *Cand. Dojkabacteria* (1) (Figure 3, Table 1 and Supplementary Figures 3–6). For four SAGs, not all three parallel phylogenetic analyses could be performed due to either missing full-length 16S rRNA gene sequences, limited completeness or a lack of closely related and publicly available reference genomes of sufficient quality (Table 1).

The observed GC content values are well in line with those of published reference genomes of the same general taxa available from the NCBI database. Despite most of the SAGs and CAGs having relatively low GC contents (Table 1), which would indicate a possible systematic MDA bias against high GC genome regions, they are not significantly lower than what would be expected from the reference genomes, which are, however, mostly represented by MAGs (Supplementary Table 5). Instead, the low GC values are likely to related to the assumed symbiotic lifestyle of Patescibacteria/CPR members (Mann and Chen, 2010).

In order to determine the novelty of the here derived SAGs and CAGs with respect to their relatedness to already existing genomes in public databases, the genomes were incorporated into the GTDB taxonomy (Parks et al., 2020). They were also directly compared to their most closely related references (Figure 3) regarding ANI and average amino acid identity (AAI) values (Kim et al., 2014; Luo et al., 2014). The combined results suggest that the closest known neighbors of the SAGs and CAGs belong to a different species (in seven cases), a different genus (in three cases) or even a different family (also in three cases) (Table 1). However, for the two most incomplete SAGs the distance to the closest neighbors could not be reliably determined. Since only genomes available from NCBI and of sufficient quality were used for the analysis, it is of course possible that related cells could not be assigned, most likely also due to low completeness values. For those CAGs and SAGs with their closest neighbors being part of this study, it was found that CAG2 and CAG3 both do belong to the same novel family of *Cand. Komeilibacteria*, but not to the same genus. The closely related novel *Cand. Shapirobacteria* S1_L14, S2_F12, S3_E17, and S4_G10 are all novel species within the same genus, while S1_L17 constitutes a novel genus and S1_J13 is a novel species in an already represented genus. On 16S rRNA gene-level, the sequence identities to already deposited 16S rRNA genes from amplicon and clone studies lie between 81.7 and 97.5%. Interestingly, these data also support that the new SAGs and CAGs constitute new species, genera and families that where not found before (Yarza et al., 2014), despite the large amount of environmental 16S rRNA gene sequences available.

It has been described previously that SAGs, although usually less complete than MAGs, contain regions derived by horizontal gene transfer that are not binned by metagenomic approaches (Dick et al., 2009; Dam et al., 2020). In this study, 21 MAGs of Candidatus taxa, including 19 Patescibacteria/CPR could be retrieved from the metagenomic dataset. However, only one CAG (CAG1) and no SAGs could be unambiguously attributed to a MAG of the same species. This discrepancy of findings distinctly highlights the importance of combining metagenomic and single-cell analyses for the analysis of complex environmental samples. The matching CAG was found to contain about 0.23 Mb (31.4%) of sequence information spread across 427 genomic regions ranging between 10 kb and 200 bp in size, which are not present in the corresponding MAG (Supplementary Table 6). However, almost none of the genes encoded within these regions could be functionally annotated beyond “hypothetical protein.” While this makes it impossible to verify any involvement of horizontal gene transfer events or genome plasticity, it reflects that the reference data basis for Patescibacteria/CPR annotation is much lower than for other taxonomic groups and is moreover predominantly represented by metagenomic bins that would likely lack exactly such laterally acquired regions. This underlines the need for more studies on this phylum that are not only relying on MAGs but also try to capture more SAGs or even try to cultivate Patescibacteria/CPR.

When focusing on the genomic regions with more differentiated annotation results, it becomes obvious that many of the proteins are predominantly related to essential cell functions like cell wall biogenesis, protein biosynthesis, replication, and—to a lesser extent—carbohydrate metabolism (Supplementary Figure 7). Especially the latter seems noteworthy as no proteins involved in the tricarboxylic acid cycle (TCA) and no cytochromes involved in oxygen metabolism were found in any SAG. This is not unheard of as Patescibacteria/CPR was inferred to be non-respiring before (Wrighton et al., 2012; Castelle et al., 2017), however, it should be noted that the lack of certain genes could be caused by the incompleteness of the genomes. While all genes of the glycolysis could be found in at least three instances each, the 6-phosphofructokinase gene was not detectable in any genome, indicating that glycolysis might also not be functional in the analyzed Patescibacteria/CPR. This gene can often be found missing in Patescibacteria/CPR genomes, but in this case neither a suggested bypass through the non-oxidative pentose phosphate pathway (PPP) (Kantor et al., 2013; Jaffe et al., 2020) nor a usage of the Entner–Doudoroff pathway could be inferred as no genes encoding these pathways were detected. A possible way of energy and/or resources acquisition might be the usage of an archaeal form III ribulose-1,5-biphosphate carboxylase/oxygenase (RuBisCO) (encoded in two SAGs) (Wrighton et al., 2016). This way, 3-phosphoglycerate (derived from CO_2_ and ribulose-1,5-bisphosphate) might be channeled in the central metabolism where it could be used as substrate for fermentation, for example lactic acid fermentation with lactate dehydrogenase being found in one SAG. The 3-phosphoglycerate might also be used in gluconeogenesis, as all necessary genes for this pathway were present—it is, however, not clear which further way products of this pathway might take. However, the presence of organisms with this metabolic focus makes sense in the context of a viticultural wastewater treatment plant with high supply of sugars but undependable supply of oxygen.

## Conclusion

Our study gives a first insight into the opportunities derived from sorting environmental unlabeled single cells with a novel commercial instrument. The extremely successful MDA yield of more than 80% shows that many cells are accessible to WGA when no harmful labeling techniques are employed, and only moderate pressure is applied. The thereby gathered single cell- and co-assembled genomes are a valuable resource for extending the knowledge on the Patescibacteria/CPR. While currently 3,128 Patescibacteria/CPR genomes are available (November 2020, GOLD database), only 745 are derived from single cells while the majority are MAGs, and only oral *Cand. Saccharibacteria* are available in co-culture. Despite the MAGs having several obvious advantages, they e.g., severely limit the possibility to identify horizontally derived or otherwise ambiguous genome parts, do not resolve heterogeneities between close relatives and do not allow 16S rRNA gene-based analyses. Therefore, a combination of MAGs with still underrepresented SAG data would be desirable to further approach elusive microbial dark matter such as the Patescibacteria/CPR. In future studies, the single-cell printer’s gentle handling of the cells and lack of staining requirement would also be very beneficial for transcriptomic as well as cultivation approaches, especially in the light of no pure culture of Patescibacteria/CPR being available. In that context, further big advantages of the instrument are the relatively compact size and low required sample/buffer volumes (about 40 μl), potentially enabling relatively easy anaerobic sorts simply by placing it within an anaerobic glove box, which requires far more complicated procedures when using FACS because large volumes of sheath fluids [typically several liters (Rinke et al., 2014)] would need to be kept oxygen-free as well (Thompson et al., 2015).

In this study, 27 SAGs were derived from a single cell but 20 of the analyzed WGAs showed indications of containing genome fragments from two or more different organisms. While this effect is mostly likely explainable by error margins during cell detection occurring with any sorting technique, the here used single-cell printer method has a distinct prominent feature that may prove highly advantageous in this context: the automized generation of micrographs of each sorting event. While micrograph generation has already been incorporated into previous cell sorting workflows (Woyke et al., 2010; Dodsworth et al., 2013), these usually required custom set-ups and manual manipulation, restricting the throughput of such techniques. In case of the ambiguous SAGs, such images could be employed to retrospectively elucidate whether multiple cells were included in one drop and if they were attached or independent of each other. Eventually, the micrographs do not only bear the potential to assess the quality of the sorting and to gather morphological information, such as cell form and size, but they could conceivably also be used to continuously refine the single-cell printers’ detection models. This in turn might lead to a more specific application of WGA and sequencing which would allow to reduce experimental costs. Not only for these reasons, it might also be favorable to increase camera resolutions in future models to further fit microbiologists’ needs. Furthermore, such micrographs do not only enable verification of the separation status of the gained single cells but potentially could also allow directly targeting attached cells, e.g., for studies of cell-cell association. In addition, the instrument can be equipped with a laser system, then also allowing the sorting of targeted cells. Here, also multiple cells with the same feature could be sorted in one well, which might be beneficial for certain biological questions. The protocol could even be combined with a fluorescent label to create a phylogenetically or functionally targeted approach.

We have shown for the first time that single-cell printing is a successful and expandable method to examine microbial dark matter from environmental samples that will allow for very diverse future applications in the fields of genomics, transcriptomics and culturomics. Moreover, the unique cell detection technique *via* photographic imaging represents a distinct feature of this method, which shows high potential for future microbial applications, if further improved. In summary, we were able to unravel 15 distinct genomes of Patescibacteria/CPR members with this method, representing novel species, genera and even families.

## Data Availability Statement

The datasets presented in this study can be found in online repositories. The names of the repository/repositories and accession number(s) can be found below: https://www.ncbi.nlm.nih.gov/, PRJNA664701.

## Author Contributions

HD and A-KK designed the study. HD and JR performed the experiments. SW and JV analyzed the data. SW and A-KK wrote the manuscript with help from JV and JR. All authors read and approved the final manuscript.

## Conflict of Interest

JR is an employee of cytena GmbH, a company that is developing single-cell analysis and handling solutions. The remaining authors declare that the research was conducted in the absence of any commercial or financial relationships that could be construed as a potential conflict of interest.

## Footnotes

1 https://motu-tool.org/fetchMG.html

2 http://ecogenomics.github.io/BamM

3 https://github.com/dparks1134/PhyloRank
