# Supplementary material for: Printing Microbial Dark Matter: Using Single Cell Dispensing and Genomics to Investigate the Patescibacteria/Candidate Phyla Radiation
Source: Front Microbiol. 2021 Jun 16;12:635506. doi: 10.3389/fmicb.2021.635506 (PMC8241940; doi:10.3389/fmicb.2021.635506)
Supplement: Supplementary file 1 [file Data_Sheet_2.PDF]

# Supplementary material

# Printing Microbial Dark Matter: Using Single Cell Dispensing and Genomics to Investigate the Patescibacteria/Candidate Phyla Radiation

Sandra Wiegand[1]
Hang T. Dam[1]
Julian Riba[2]
John Vollmers[1]
Anne-Kristin Kaster[1,3]

[1] Institute for Biological Interfaces 5, Karlsruhe Institute of Technology, Germany
[2] Laboratory for MEMS Applications, IMTEK - Department of Microsystems Engineering, University of Freiburg, Germany
[3] Institute for Applied Biosciences, Karlsruhe Institute of Technology, Germany

Corresponding author: kaster@kit.edu

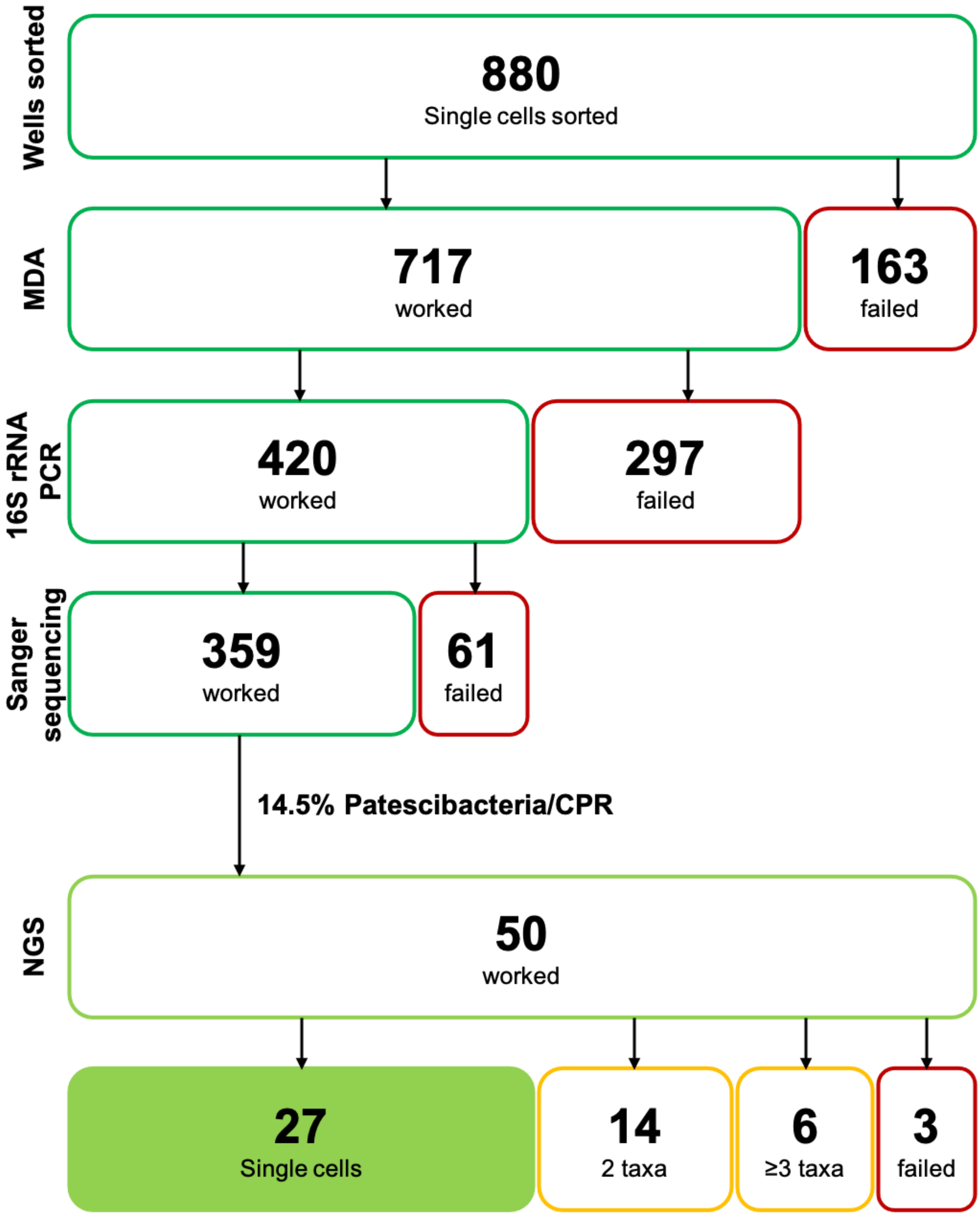


**Supplementary Figure S1: Single cell genomics workflow with single cell dispenser.** Flowchart reflecting the results described throughout the manuscript.

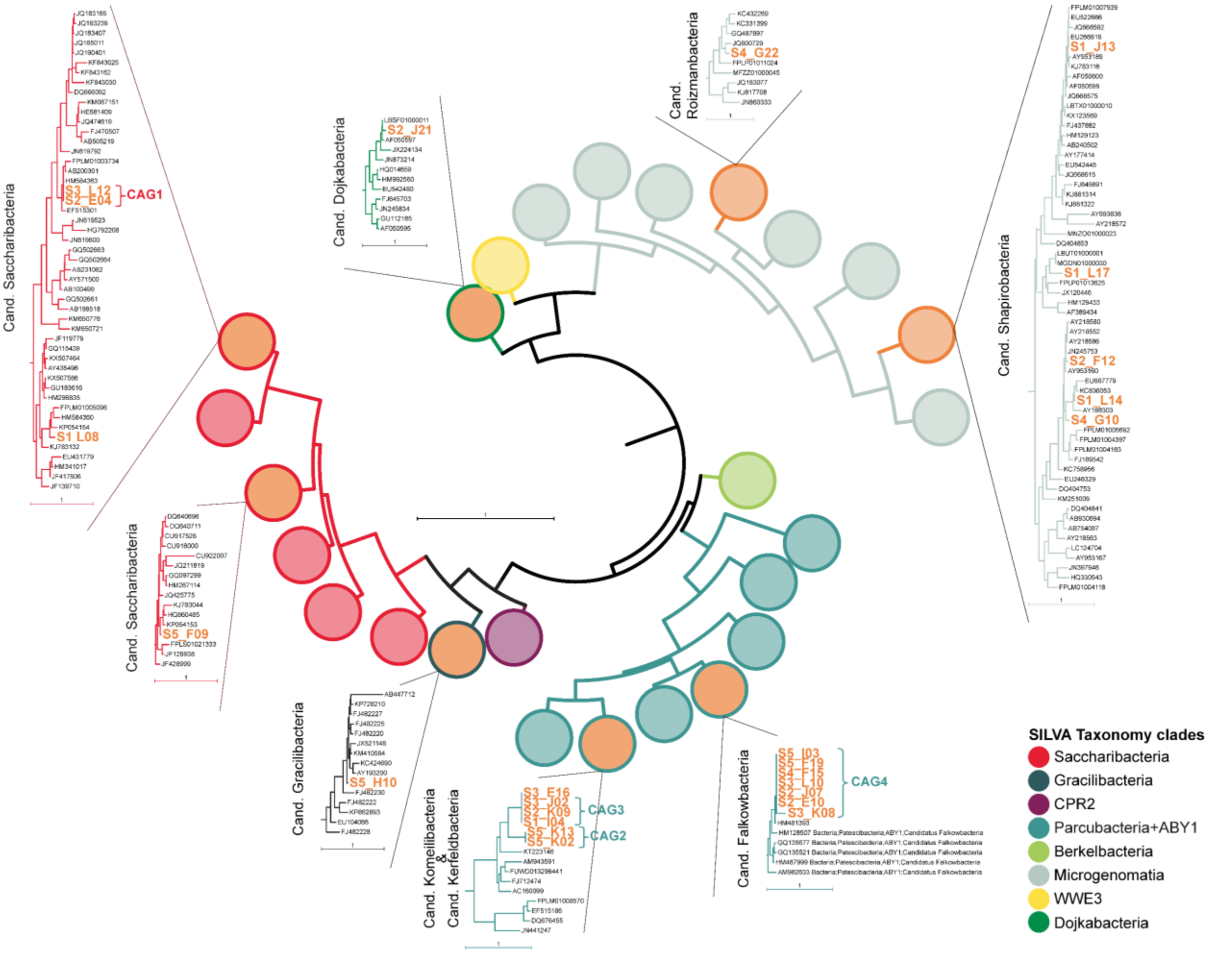


**Supplementary Figure S2: 16S rRNA-based phylogenetic assessment of SAGs and CAGs of the Patescibacteria/CPR.** The tree was calculated from the Patescibacteria/CPR branch of the SILVA SSU 138 Ref NR 99 release upon addition of the SAGs 16S rRNA sequences (Yilmaz et al., 2014). In total, the tree contains 4569 Patescibacteria/CPR sequences and 52 sequences of the not shown Poribacteria outgroup. Clades with 15 or less branches were deleted from the tree for better readability and larger clades were collapsed. The colors of the branches represent Patescibacteria/CPR subtaxa. SAGs are indicated in orange. The detailed sections are taken from the same tree to indicate the closest neighbors of the SAGs.

Yilmaz, P., Parfrey, L.W., Yarza, P., Gerken, J., Pruesse, E., Quast, C., et al. (2014). The SILVA and "All-species Living Tree Project (LTP)" taxonomic frameworks. *Nucleic Acids Res* 42**,** D643-648. doi: 10.1093/nar/gkt1209.

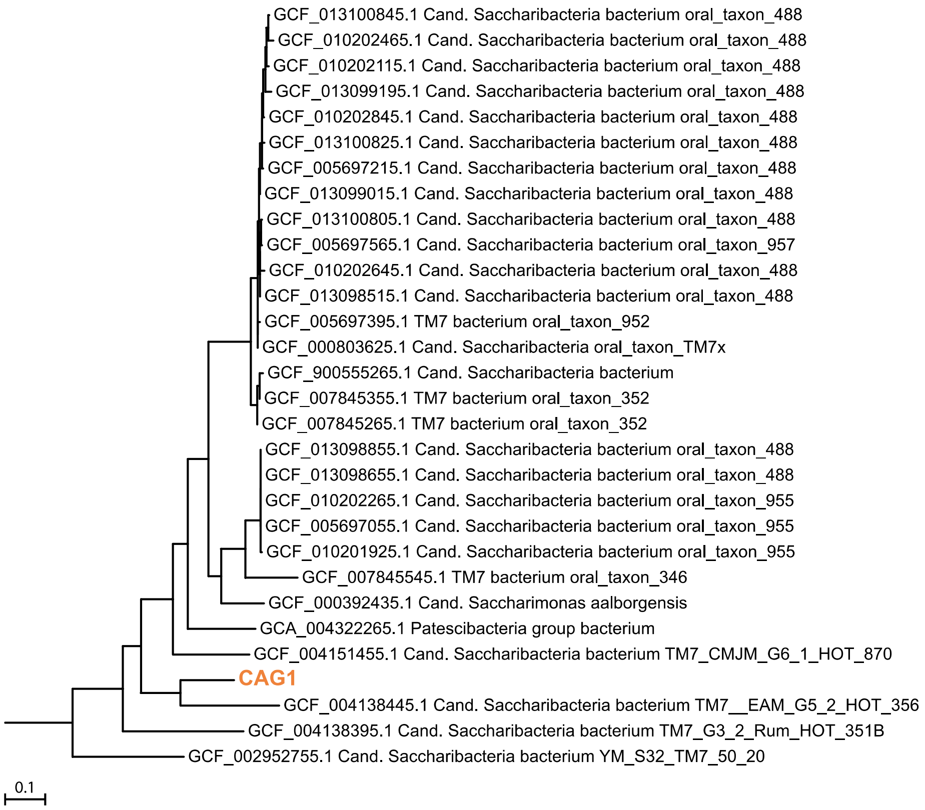


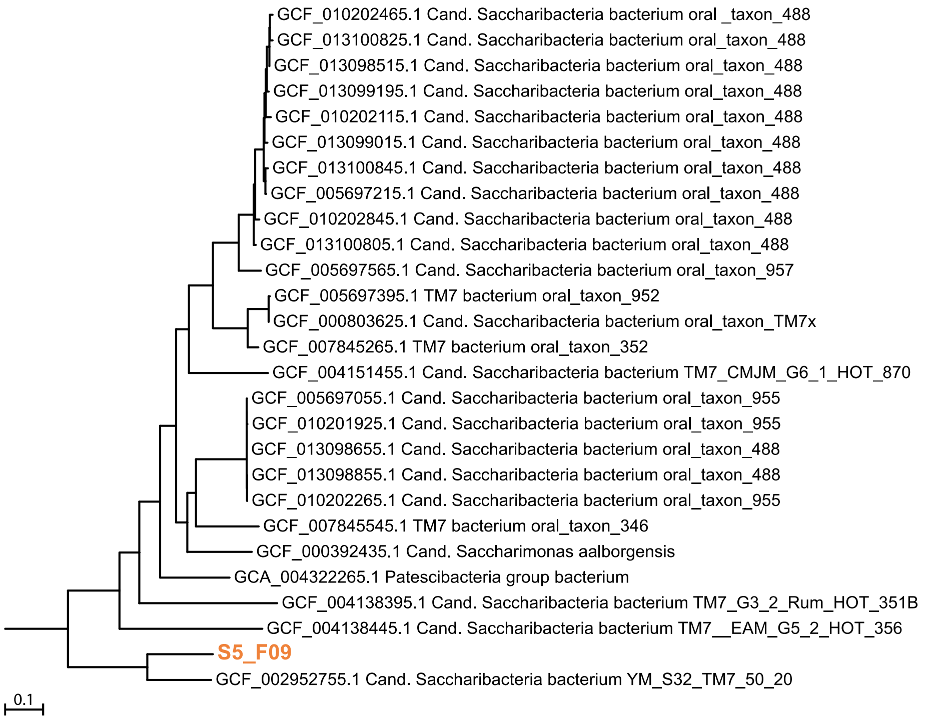


**Supplementary Figure S3: Multilocus sequence alignment based only on identified orthologous proteins – Cand. Saccharibacteria.** Orthologous genes shared between SAGs or CAGs and selected Patescibacteria/CPR reference genomes were determined via bidirectional BLAST analysis, aligned and plotted. Chloroflexi were used as outgroup. SAGs/CAGs are indicated in orange and all genomes but from the closest neighbors were removed from the tree to enhance readability.

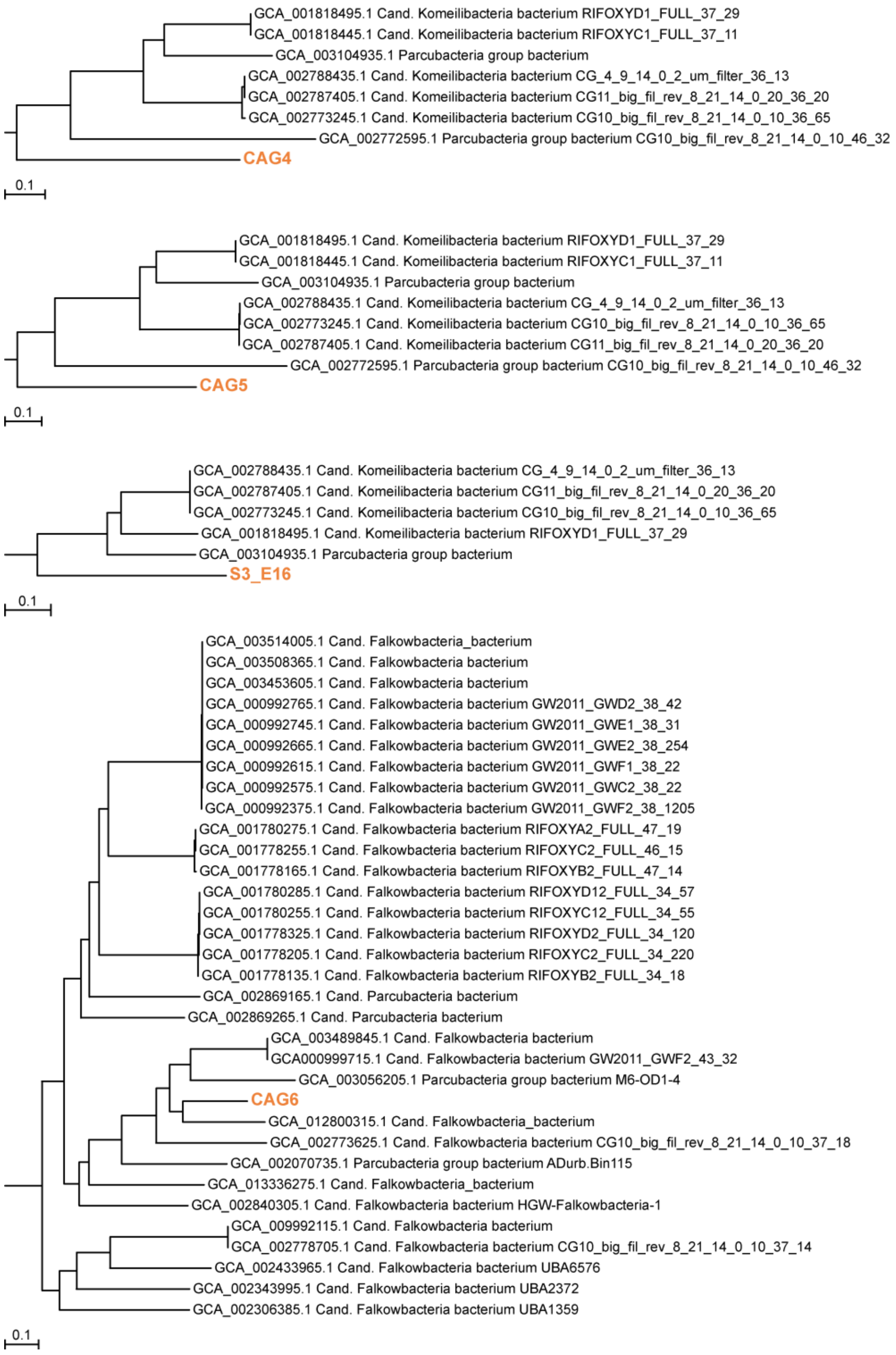


**Supplementary Figure S4: Multilocus sequence alignment based only on identified orthologous proteins – Parcubacteria/ABY1.** Orthologous genes shared between SAGs or CAGs and selected Patescibacteria/CPR reference genomes were determined via bidirectional BLAST analysis, aligned and plotted. Chloroflexi were used as outgroup. SAGs/CAGs are indicated in orange and all genomes but from the closest neighbors were removed from the tree to enhance readability.

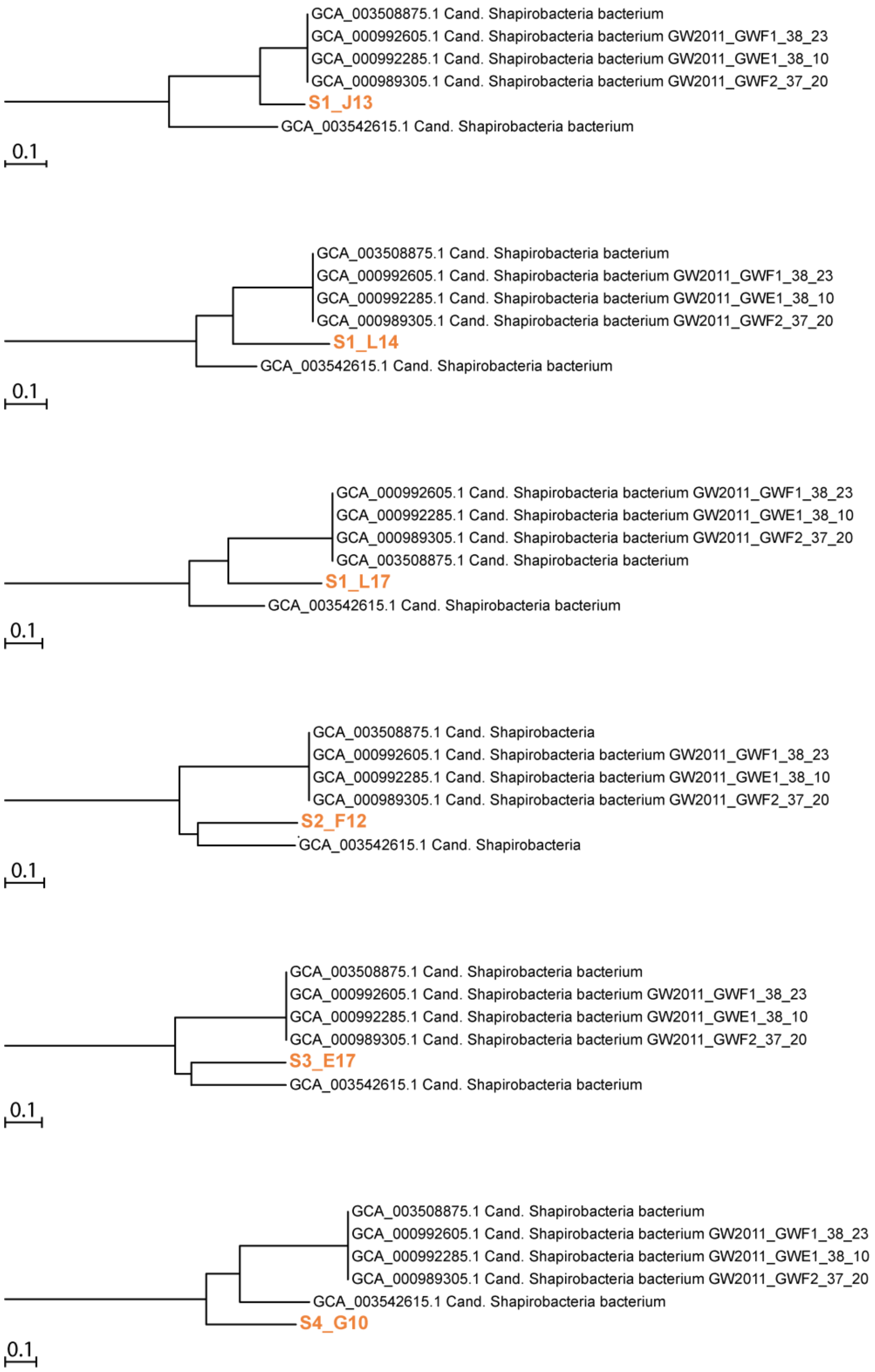


**Supplementary Figure S5: Multilocus sequence alignment based only on identified orthologous proteins – Cand. Shapirobacteria.** Orthologous genes shared between SAGs or CAGs and selected Patescibacteria/CPR reference genomes were determined via bidirectional BLAST analysis, aligned and plotted. Chloroflexi were used as outgroup. SAGs/CAGs are indicated in orange and all genomes but from the closest neighbors were removed from the tree to enhance readability.

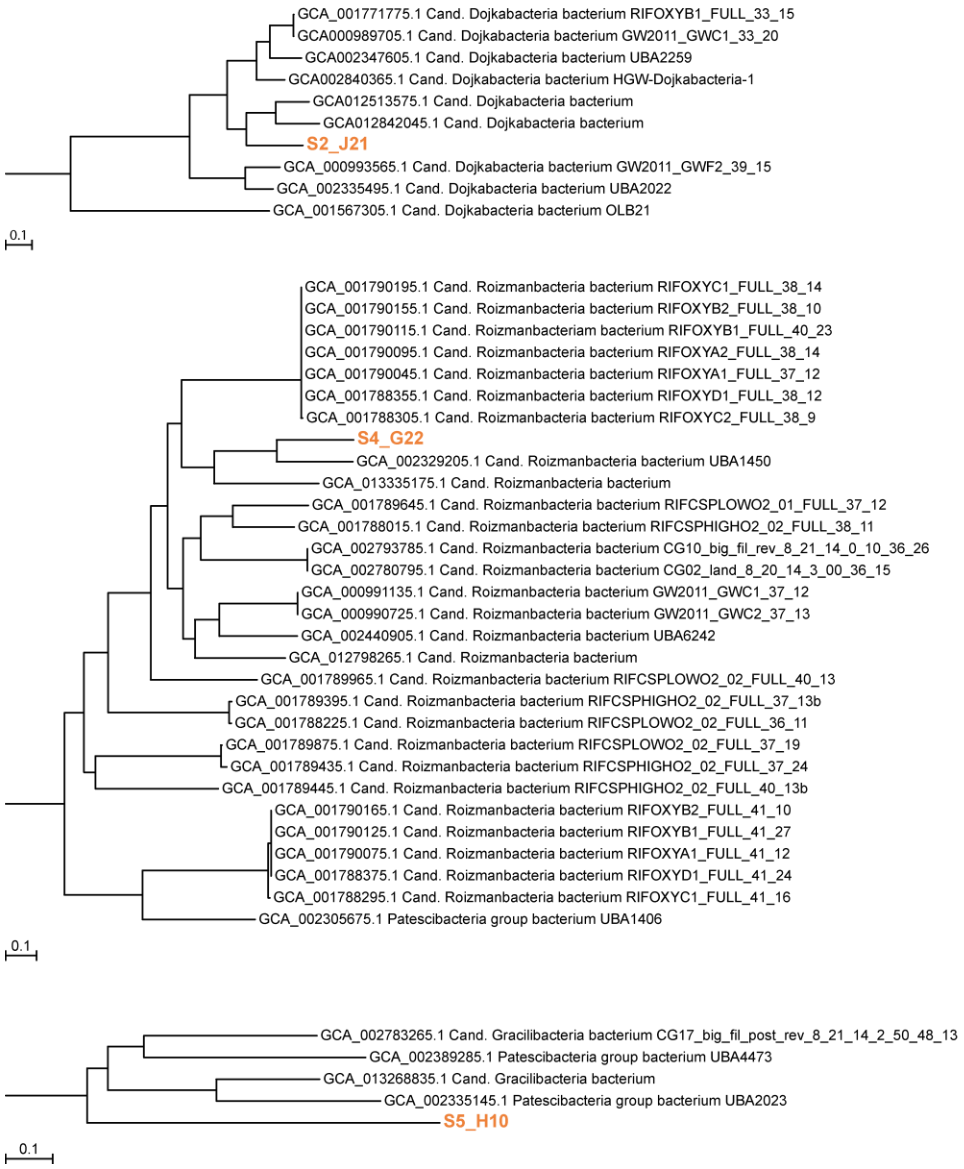


**Supplementary Figure S6: Multilocus sequence alignment based only on identified orthologous proteins – Cand. Dojkabacteria, Cand. Roizmanbacteria and Cand. Gracilibacteria.** Orthologous genes shared between SAGs or CAGs and selected Patescibacteria/CPR reference genomes were determined via bidirectional BLAST analysis, aligned and plotted. Chloroflexi were used as outgroup. SAGs/CAGs are indicated in orange and all genomes but from the closest neighbors were removed from the tree to enhance readability.

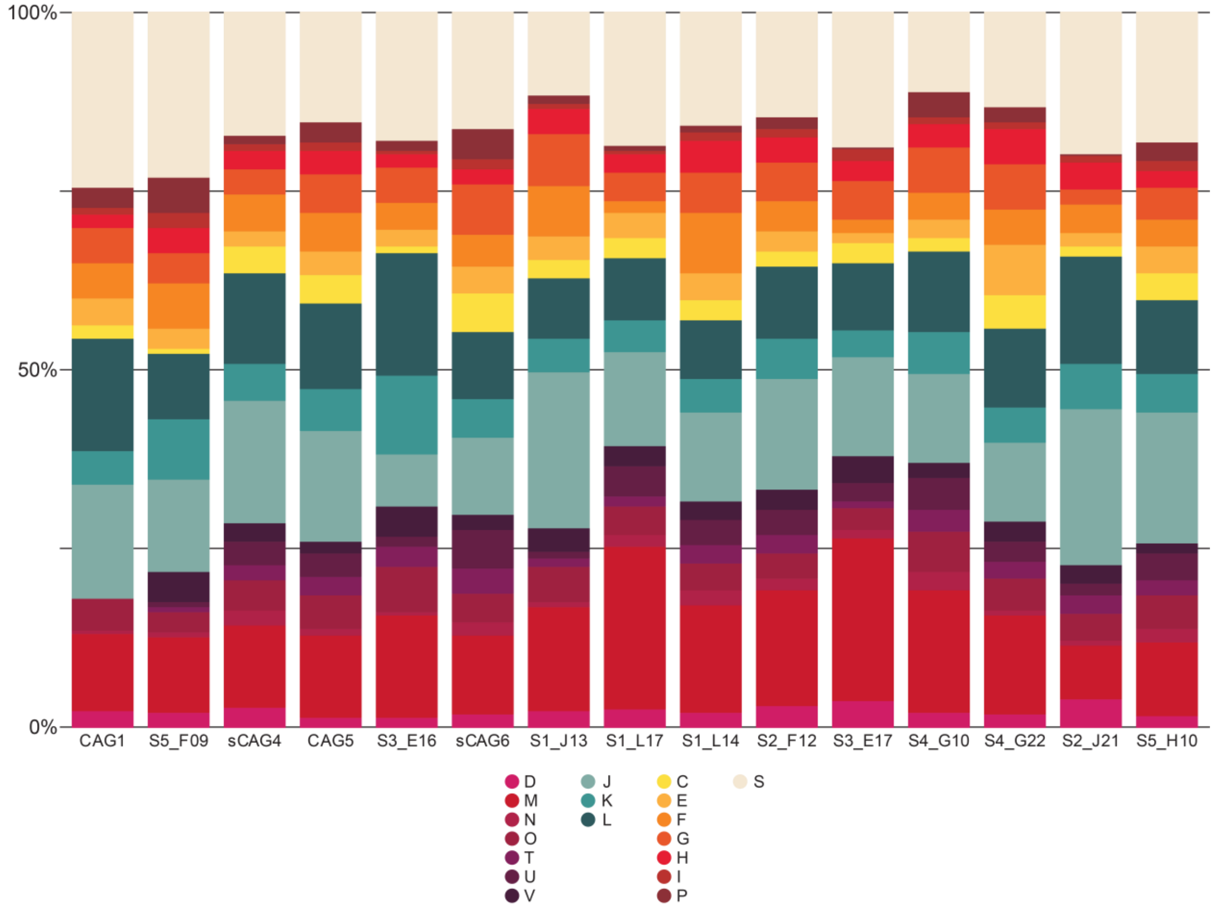


**Supplementary Figure S7: Cluster of orthologous genes (COG) analysis of unique SAGs. Cellular processes and signaling.** (D) Cell cycle control, cell division, chromosome partitioning, (M) Cell wall/membrane/envelope biogenesis, (N) Cell motility, (O) Post-translational modification, protein turnover, and chaperones, (T) Signal transduction mechanisms, (U) Intracellular trafficking, secretion, and vesicular transport, (V) Defense mechanisms. **Information storage and processing:** (J) Translation, ribosomal structure and biogenesis, (K) Transcription, (L) Replication, recombination and repair. **Metabolism:** (E) Amino acid transport and metabolism, (F) Nucleotide transport and metabolism, (G) Carbohydrate transport and metabolism, (H) Coenzyme transport and metabolism. (I) Lipid transport and metabolism, (P) Inorganic ion transport and metabolism. **Poorly characterized:** (S) Function unknown.

**Supplementary Table S1: The aerated lagoon (LEA) of a winery wastewater treatment plant - microbial community composition.**

| Phylum | Class | Order | Family | Genus | Identified marker genes |
|---|---|---|---|---|---|
| Cand. Saccharibacteria | None | None | None | None | 33727 |
| Unclassified CPR | None | None | None | None | 11176 |
| Elusimicrobia | None | None | None | None | 2743 |
| Bacteroidetes | Bacteroidia | Bacteroidales | None | None | 1351 |
| Bacteroidetes | Bacteroidia | Marinilabiliales | None | None | 221 |
| Bacteroidetes | Bacteroidia | None | None | None | 535 |
| Bacteroidetes | Flavobacteriia | Flavobacteriales | Flavobacteriaceae | None | 316 |
| Bacteroidetes | Flavobacteriia | None | None | None | 314 |
| Bacteroidetes | None | None | None | None | 8005 |
| Cand. Cloacimonetes | None | None | None | None | 1280 |
| Ignavibacteriae | None | None | None | None | 439 |
| Unclassified FCB | None | None | None | None | 393 |
| Proteobacteria | Alphaproteobacteria | None | None | None | 8731 |
| Proteobacteria | Alphaproteobacteria | Rhizobiales | None | None | 6189 |
| Proteobacteria | Alphaproteobacteria | Rhodobacterales | Rhodobacteraceae | None | 2692 |
| Proteobacteria | Alphaproteobacteria | Rhodobacterales | Rhodobacteraceae | Rhodobacter | 295 |
| Proteobacteria | Betaproteobacteria | Burkholderiales | Comamonadaceae | None | 225 |
| Proteobacteria | Betaproteobacteria | Burkholderiales | None | None | 847 |
| Proteobacteria | Betaproteobacteria | None | None | None | 3660 |
| Proteobacteria | Betaproteobacteria | Rhodocyclales | None | None | 203 |
| Proteobacteria | Betaproteobacteria | Rhodocyclales | Zoogloeaceae | None | 241 |
| Proteobacteria | Deltaproteobacteria | Desulfuromonadales | None | None | 490 |
| Proteobacteria | Deltaproteobacteria | None | None | None | 385 |
| Proteobacteria | Epsilonproteobacteria | Campylobacterales | Campylobacteraceae | None | 429 |
| Proteobacteria | Gammaproteobacteria | None | None | None | 348 |
| Proteobacteria | Gammaproteobacteria | Xanthomonadales | None | None | 965 |
| Proteobacteria | None | None | None | None | 1259 |
| Planctomycetes | None | None | None | None | 346 |
| Unclassified PVC | None | None | None | None | 614 |
| Verrucomicrobia | None | None | None | None | 1270 |
| Verrucomicrobia | Opitutae | None | None | None | 288 |
| Actinobacteria | Actinobacteria | Actinomycetales | Actinomycetaceae | None | 914 |
| Actinobacteria | Actinobacteria | Micrococcales | None | None | 1388 |
| Actinobacteria | Actinobacteria | None | None | None | 6034 |
| Actinobacteria | Actinobacteria | Propionibacteriales | None | None | 18225 |
| Actinobacteria | Actinobacteria | Propionibacteriales | Propionibacteriaceae | None | 23977 |
| Actinobacteria | Actinobacteria | Propionibacteriales | Propionibacteriaceae | Tessaracoccus | 1828 |
| Actinobacteria | None | None | None | None | 23410 |
| Chloroflexi | None | None | None | None | 1428 |
| Firmicutes | Clostridia | Clostridiales | None | None | 604 |
| Firmicutes | Clostridia | None | None | None | 289 |
| Firmicutes | None | None | None | None | 2805 |
| Unclassified Terrabacteria | None | None | None | None | 58885 |
| Spirochaetes | None | None | None | None | 3614 |
| Spirochaetes | Spirochaetia | Spirochaetales | Spirochaetaceae | Treponema | 310 |
| Unclassified | None | None | None | None | 57707 |

**Supplementary Table S2: Ratio of cellular DNA to free DNA extracted from 1ml source material before and after bulk sorting (> 5 Million Cells) via FACS.** Parallels of 1 ml each of *E. coli* culture was supplemented with glycerol-TE. For soil samples, 2 g of soil were resuspended in PBS and briefly centrifuged at low speeds in order to remove large particles. Glycerol-TE was added to the supernatant to a final concentration of 15%, which was then aliquoted into 1 ml portions. Both sample types were frozen at -80 °C according to common practice for samples for cell sorting. For sorting, 1 ml of sample was ~5x diluted with PBS, and sorted yielding roughly 5 million cells into a final resulting volume of 5 ml. For both sample types, tests were done in duplicate with one unsorted and one sorted sample being tested each. For each test, the cellular fraction was pelleted via centrifugation at 17.000 x *g* for 5 minutes, after which pellet and supernatant were separated. DNA was extracted from the cellular fraction using the MPBio FastDNA Extraction kit for soil (MP Biomedicals, Eschwege, Germany), while free DNA was precipitated from the supernatant via alcohol precipitation. DNA content was measured using a fluorometric assay, specific for double stranded DNA (Qbit DNA HS).

| | **Unsorted** | | | **FACS** | | |
|---|---|---|---|---|---|---|
| | **DNA content pellet** | **DNA content supernatant** | **Ratio cellular to free DNA** | **DNA content pellet** | **DNA content supernatant** | **Ratio cellular to free DNA** |
| ***E. coli* 1** | 2240 ng | 427 ng | 5.2 x | 1.2 ng | 1.1 ng | 1 x |
| ***E. coli* 2** | 2080 ng | 179 ng | 11.6 x | 2.4 ng | 4.2 ng | 0.6 x |
| **Soil sample 1** | 44.8 ng | 0 ng | NaN | 0.0 ng | 8.9 ng | 0 x |
| **Soil sample 2** | 38.9 ng | 8.2 ng | 4.7 x | 0.0 ng | 6,3 ng | 0 x |

**Supplementary Table S3: The aerated lagoon (LEA) of a winery wastewater treatment plant - taxonomic composition of cells after cell sorting based on 16S Sanger sequencing.**

| **Phylum** | **Class** | **Order** | **Family** | **Genus** | **Sanger reads** |
|---|---|---|---|---|---|
| Proteobacteria | Gammaproteobacteria | Legionellales | Legionellaceae | Legionella | 1 |
| Proteobacteria | Gammaproteobacteria | Methylococcales | None | None | 1 |
| Proteobacteria | Gammaproteobacteria | None | None | None | 1 |
| Proteobacteria | Gammaproteobacteria | Pseudomonadales | Pseudomonadaceae | Pseudomonas | 5 |
| Proteobacteria | Betaproteobacteria | Burkholderiales | Alcaligenaceae | None | 5 |
| Proteobacteria | Betaproteobacteria | Burkholderiales | Comamonadaceae | Giesbergeria | 1 |
| Proteobacteria | Betaproteobacteria | Burkholderiales | Comamonadaceae | Hydrogenophaga | 16 |
| Proteobacteria | Betaproteobacteria | Burkholderiales | Comamonadaceae | None | 88 |
| Proteobacteria | Betaproteobacteria | Burkholderiales | None | None | 3 |
| Proteobacteria | Betaproteobacteria | Ferritrophicales | Ferritrophicaceae | Ferritrophicum | 1 |
| Proteobacteria | Betaproteobacteria | Neisseriales | Neisseriaceae | None | 20 |
| Proteobacteria | Betaproteobacteria | Nitrosomonadales | Methylophilaceae | None | 1 |
| Proteobacteria | Betaproteobacteria | Rhodocyclales | Rhodocyclaceae | None | 2 |
| Proteobacteria | Alphaproteobacteria | Caulobacterales | Caulobacteraceae | Brevundimonas | 1 |
| Proteobacteria | Alphaproteobacteria | None | None | None | 3 |
| Proteobacteria | Alphaproteobacteria | Rhizobiales | None | None | 2 |
| Proteobacteria | Alphaproteobacteria | Rhodobacterales | Rhodobacteraceae | None | 4 |
| Proteobacteria | Alphaproteobacteria | Rhodospirillales | Acetobacteraceae | Roseomonas | 1 |
| Proteobacteria | Epsilonproteobacteria | Campylobacterales | None | None | 4 |
| Proteobacteria | Epsilonproteobacteria | None | None | Sulfurovum | 5 |
| Cand. Dependentiae | Candidatus Babeliae | Candidatus Babeliales | None | None | 4 |
| Spirochaetes | Spirochaetia | Leptospirales | Leptospiraceae | None | 2 |
| Spirochaetes | Spirochaetia | Spirochaetales | Spirochaetaceae | None | 4 |
| Spirochaetes | Spirochaetia | Spirochaetales | Spirochaetaceae | Sphaerochaeta | 5 |
| Spirochaetes | Spirochaetia | Spirochaetales | Spirochaetaceae | Treponema | 1 |
| Proteobacteria | Deltaproteobacteria | Desulfobacterales | Desulfobulbaceae | Desulfobulbus | 1 |
| Proteobacteria | Deltaproteobacteria | Desulfobacterales | None | None | 3 |
| Proteobacteria | Deltaproteobacteria | Desulfovibrionales | Desulfomicrobiaceae | Desulfomicrobium | 1 |
| Proteobacteria | Deltaproteobacteria | Desulfuromonadales | Geobacteraceae | Geobacter | 1 |
| Proteobacteria | Deltaproteobacteria | Desulfuromonadales | Geobacteraceae | None | 2 |
| Elusimicrobia | Elusimicrobia | None | None | None | 1 |
| Chlamydiae | Chlamydiia | Chlamydiales | Chlamydiaceae | None | 1 |
| Chlamydiae | Chlamydiia | Parachlamydiales | Parachlamydiaceae | None | 1 |
| Kiritimatiellaeota | Kiritimatiellae | None | None | None | 2 |
| Planctomycetes | None | None | None | None | 3 |
| Planctomycetes | Planctomycetia | Planctomycetales | Isosphaeraceae | None | 1 |
| Planctomycetes | Planctomycetia | Planctomycetales | None | None | 2 |
| Planctomycetes | Planctomycetia | Planctomycetales | Planctomycetaceae | Blastopirellula | 1 |
| Planctomycetes | Planctomycetia | Planctomycetales | Planctomycetaceae | Planctopirus | 1 |
| Verrucomicrobia | Opitutae | Opitutales | Opitutaceae | Lacunisphaera | 1 |
| Verrucomicrobia | Spartobacteria | None | None | Terrimicrobium | 1 |
| Verrucomicrobia | Verrucomicrobiae | Verrucomicrobiales | Verrucomicrobiaceae | Luteolibacter | 1 |
| Verrucomicrobia | Verrucomicrobiae | Verrucomicrobiales | Verrucomicrobiaceae | Prosthecobacter | 1 |
| Bacteroidetes | Bacteroidia | Bacteroidales | Lentimicrobiaceae | None | 3 |
| Bacteroidetes | Bacteroidia | Bacteroidales | None | None | 1 |
| Bacteroidetes | Bacteroidia | Bacteroidales | Paludibacteraceae | None | 1 |
| Bacteroidetes | Bacteroidia | Bacteroidales | Rikenellaceae | None | 25 |
| Bacteroidetes | Bacteroidia | Bacteroidales | Tannerellaceae | None | 1 |
| Bacteroidetes | Bacteroidia | Marinilabiliales | Prolixibacteraceae | None | 7 |

| | | | | | |
|---|---|---|---|---|---|
| Bacteroidetes | Bacteroidia | None | None | None | 2 |
| Bacteroidetes | Chitinophagia | Chitinophagales | Chitinophagaceae | Edaphobaculum | 10 |
| Bacteroidetes | Chitinophagia | Chitinophagales | Chitinophagaceae | None | 1 |
| Bacteroidetes | Cytophagia | Cytophagales | Cytophagaceae | Dyadobacter | 1 |
| Bacteroidetes | Flavobacteriia | Flavobacteriales | Crocinitomicaceae | Fluviicola | 10 |
| Bacteroidetes | Flavobacteriia | Flavobacteriales | Crocinitomicaceae | None | 5 |
| Bacteroidetes | Flavobacteriia | Flavobacteriales | Flavobacteriaceae | Flavobacterium | 8 |
| Bacteroidetes | Saprospiria | Saprospirales | Saprospiraceae | None | 11 |
| Bacteroidetes | Sphingobacteriia | Sphingobacteriales | None | None | 1 |
| Bacteroidetes | Sphingobacteriia | Sphingobacteriales | Sphingobacteriaceae | Pedobacter | 2 |
| Balneolaeota | Balneolia | Balneolales | Balneolaceae | None | 4 |
| Cand. Cloacimonetes | None | None | None | None | 7 |
| Cand. Kapabacteria | None | None | None | None | 1 |
| Synergistetes | Synergistia | Synergistales | Synergistaceae | None | 1 |
| Actinobacteria | Actinobacteria | Propionibacteriales | Propionibacteriaceae | Cutibacterium | 1 |
| Actinobacteria | Actinobacteria | Propionibacteriales | Propionibacteriaceae | None | 3 |
| Chloroflexi | Anaerolineae | Anaerolineales | Anaerolineaceae | None | 3 |
| Firmicutes | Bacilli | Lactobacillales | Carnobacteriaceae | None | 7 |
| Firmicutes | Bacilli | Lactobacillales | Carnobacteriaceae | Trichococcus | 21 |
| Firmicutes | Bacilli | None | None | None | 3 |
| Firmicutes | Clostridia | Clostridiales | Christensenellaceae | None | 3 |
| Firmicutes | Clostridia | Clostridiales | Clostridiales Family XII. Incertae Sedis | Fusibacter | 1 |
| Firmicutes | Clostridia | Clostridiales | Eubacteriaceae | Acetobacterium | 1 |
| Firmicutes | Clostridia | Clostridiales | Hungateiclostridiaceae | None | 2 |
| Firmicutes | Clostridia | Clostridiales | Oscillospiraceae | None | 1 |
| Firmicutes | Clostridia | Clostridiales | Peptococcaceae | None | 1 |
| Firmicutes | Clostridia | None | None | None | 5 |
| Firmicutes | Negativicutes | Selenomonadales | Sporomusaceae | None | 2 |
| Firmicutes | None | None | None | None | 2 |
| Cand. Nomurabacteria | None | None | None | None | 1 |
| Cand. Falkowbacteria | None | None | None | None | 9 |
| Cand. Kerfeldbacteria | None | None | None | None | 13 |
| Cand. Gracilibacteria | None | None | None | None | 2 |
| Cand. Saccharibacteria | None | None | None | None | 10 |
| Cand. Shapirobacteria | None | None | None | None | 14 |
| Cand. Pacebacteria | None | None | None | None | 1 |
| Cand. Roizmanbacteria | None | None | None | None | 1 |
| Cand. Dojkabacteria | None | None | None | None | 1 |

**Supplementary Table S4: ANI values within the given lineages that were used to determine which SAGs were merged to CAGs.** ANI values are given in percent (%) and are printed in bold. Corresponding alignment fraction (AF) values are given in parentheses

***Cand. Saccharibacteria***

| | S3_L12 | S2_E04 | S5_F09 | |
|---|---|---|---|---|
| **S3_L12** | **100.0** (100) | **99.9** (21.3) | **91.8** (3.4) | CAG1 |
| **S2_E04** | **99.9** (33.5) | **100.0** (100) | **86.9** (3.6) | |
| **S5_F09** | **91.8** (1.8) | **86.9** (4.3) | **100.0** (100) | |

***Cand. Komeilibacteria***

| | S1_I04 | S3_J02 | S2_K09 | S5_K02 | S5_K13 | S1_H12 | |
|---|---|---|---|---|---|---|---|
| **S1_I04** | **100** (100) | **99.7** (27.4) | **99.9** (22.4) | **79.8** (11.6) | **74.2** (12.7) | **76.1** (10.8) | CAG3 |
| **S3_J02** | **99.7** (31.7) | **100** (100) | **99.7** (39.5) | **88.8** (29.5) | **77.7** (20.1) | **84.1** (23.6) | |
| **S2_K09** | **99.9** (17.6) | **99.7** (26.8) | **100** (100) | **85.7** (23.0) | **75.9** (19.9) | **82.1** (17.0) | |
| **S5_K02** | **79.8** (7.8) | **88.8** (17.0) | **85.7** (19.6) | **100** (100) | **99.9** (58.2) | **99.9** (34.6) | CAG2 |
| **S5_K13** | **74.2** (7.1) | **77.7** (9.6) | **75.9** (14.0) | **99.9** (48.1) | **100** (100) | **100** (41.0) | |
| **S1_H12** | **76.1** (8.7) | **84.1** (16.3) | **82.1** (17.34) | **99.9** (41.5) | **100** (59.5) | **100** (100) | |

**Cand. Falkowbacteria**

| | S2_J07 | S2_E10 | S5_F19 | S5_I03 | S3_K08 | S4_F15 | S1_L12 | S3_L10 | |
|---|---|---|---|---|---|---|---|---|---|
| **S2_J07** | **100** (100) | **100** (23.3) | **99.6** (32.8) | **99.8** (49.4) | **99.6** (48.1) | **98.4** (18.3) | **99.3** (21.1) | **98.8** (23.8) | CAG4 |
| **S2_E10** | **100** (5.6) | **100** (100) | **99.9** (22.2) | **100** (37.0) | **99.9** (25.1) | **99.5** (11.9) | **99.9** (20.8) | **99.9** (12.0) | |
| **S5_F19** | **99.6** (12.0) | **99.9** (22.2) | **100** (100) | **100** (66.4) | **99.9** (43.2) | **99.8** (17.7) | **100** (37.2) | **100** (21.0) | |
| **S5_I03** | **99.8** (9.5) | **100** (29.7) | **100** (35.0) | **100** (100) | **99.9** (47.0) | **99.8** (20.1) | **100** (30.5) | **99.7** (22.4) | |
| **S3_K08** | **99.6** (13.0) | **99.9** (28.2) | **99.9** (31.8) | **99.9** (65.8) | **100** (100) | **99.6** (19.5) | **99.9** (35.1) | **99.7** (24.8) | |
| **S4_F15** | **98.4** (11.6) | **99.5** (31.7) | **99.8** (30.8) | **99.8** (66.4) | **99.6** (46.1) | **100** (100) | **99.7** (32.0) | **100** (23.8) | |
| **S1_L12** | **99.3** (8.0) | **99.9** (32.9) | **100** (38.4) | **100** (59.8) | **99.9** (49.3) | **99.7** (19.0) | **100** (100) | **99.9** (20.2) | |
| **S3_L10** | **98.8** (14.4) | **99.9** (30.4) | **100** (35.0) | **99.7** (70.8) | **99.7** (56.0) | **100** (22.7) | **99.9** (32.6) | **100** (100) | |

***Cand. Shapirobacteria***

| | S1_J13 | S2_F12 | S1_L14 | S4_G10 | S3_E17 | S1_L17 |
|---|---|---|---|---|---|---|
| **S1_J13** | **100** (100) | **77.1** (17.0) | **68.6** (10.8) | **73.7** (11.0) | **81.3** (10.7) | **75.5** (9.3) |
| **S2_F12** | **77.1** (8.2) | **100** (100) | **71.3** (11.9) | **75.7** (13.7) | **84.9** (18.6) | **79.3** (7.7) |
| **S1_L14** | **68.6** (7.1) | **71.3** (16.2) | **100** (100) | **71.6** (12.0) | **71.9** (9.7) | **68.3** (6.2) |
| **S4_G10** | **73.7** (9.3) | **75.7** (23.9) | **71.6** (15.4) | **100** (100) | **75.5** (14.3) | **67.8** (8.0) |
| **S3_E17** | **81.3** (7.0) | **84.9** (25.2) | **71.9** (9.7) | **75.5** (11.1) | **100** (100) | **88.2** (11.0) |
| **S1_L17** | **75.5** (8.2) | **79.3** (14.2) | **68.3** (8.4) | **67.8** (8.5) | **88.2** (8.1) | **100** (100) |

**Supplementary Table S5: GC content of SAGs and CAGs in the context of their respective taxa.**

| | Public database (NCBI) | | | SAGs/CAGs |
|---|---|---|---|---|
| | **Mean GC** | **Median GC** | **Range GC** | **(Range) GC** |
| **Cand. Shapirobacteria** | 37.9 | 37.8 | 37.7 – 38.4 | 30.9 – 38.7 |
| **Cand. Roizmanbacteria** | 38.7 | 37.6 | 35.8 – 52.4 | 36.7 |
| **Cand. Komeilibacteria** | 37.2 | 36.0 | 35.5 – 44.1 | 30.5 – 33.3 |
| **Cand. Falkowbacteria** | 39.8 | 38.3 | 33.0 – 48.4 | 44.4 |
| **Cand. Saccharibacteria** | 48.3 | 50.4 | 32.3 – 54.8 | 44.7 – 45.8 |
| **Cand. Gracilibacteria** | 33.3 | 28.9 | 23.9 – 53.5 | 36.2 |
| **Cand. Dojkabacteria** | 36.4 | 34.7 | 32.7 – 42.6 | 36.2 |

**Supplementary Table S6: Contigs and Contig-regions present in CAG1 but absent in the corresponding MAG1.** The complete sequence of the MAG is given at https://zenodo.org/record/4659037#.YGb-zD9CRhE.

| Contig(s) | Region | size |
|---|---|---|
| gnl\|KIT\|sCAG1__1 | 938-9077 | 8141 bp |
| | 18850-21428 | 2580 bp |
| | 30813-37394 | 6583 bp |
| | 49249-50181 | 934 bp |
| gnl\|KIT\|sCAG1__10 | 10883-11807 | 926 bp |
| gnl\|KIT\|sCAG1__13 | 431-811 | 382 bp |
| gnl\|KIT\|sCAG1__14 | 1-4212 | 4211 bp |
| gnl\|KIT\|sCAG1__141 | 1-247 | 247 bp |
| gnl\|KIT\|sCAG1__143 | 1-273 | 273 bp |
| gnl\|KIT\|sCAG1__16 | 1-318 | 318 bp |
| | 5963-6363 | 402 bp |
| gnl\|KIT\|sCAG1__17 | 2309-4141 | 1834 bp |
| gnl\|KIT\|sCAG1__2 | 6479-9143 | 2666 bp |
| | 16770-17022 | 254 bp |
| | 22622-22889 | 269 bp |
| | 26432-26841 | 411 bp |
| gnl\|KIT\|sCAG1__23 | 1-3896 | 3896 bp |
| gnl\|KIT\|sCAG1__3 | 9017-9438 | 423 bp |
| | 17219-17651 | 434 bp |
| gnl\|KIT\|sCAG1__39 | 1231-2252 | 1322 bp |
| gnl\|KIT\|sCAG1__4 | 15287-17394 | 2109 bp |
| | 31532-35820 | 4289 bp |
| gnl\|KIT\|sCAG1__41 | 1-871 | 871 bp |
| gnl\|KIT\|sCAG1__47 | 1-302 | 301 bp |
| gnl\|KIT\|sCAG1__48 | 1-202 | 202 bp |
| gnl\|KIT\|sCAG1__5 | 1490-1929 | 441 bp |
| | 15664-18079 | 2417 bp |
| | 26373-26609 | 238 bp |
| gnl\|KIT\|sCAG1__50 | 476-1085 | 1085 bp |
| gnl\|KIT\|sCAG1__52 | 1-1286 | 1286 bp |
| gnl\|KIT\|sCAG1__6 | 20107-21266 | 1161 bp |
| | 23111-23354 | 245 bp |
| | 25671-25875 | 206 bp |
| | 27722-28649 | 928 bp |
| gnl\|KIT\|sCAG1__7 | 4570 | 2323-4570 bp |
| | 5038 | 4662-5038 bp |
| gnl\|KIT\|sCAG1__8 | 17620-18753 | 1135 bp |
| gnl\|KIT\|sCAG1__9 | 10143-11198 | 1057 bp |
| | 11463-12395 | 934 bp |
| | 15877-16594 | 719 bp |

| | | | |
|---|---|---|---|
| gnl\|KIT\|sCAG1__18 | complete | 10197 | bp |
| gnl\|KIT\|sCAG1__19 | complete | 9377 | bp |
| gnl\|KIT\|sCAG1__20 | complete | 8579 | bp |
| gnl\|KIT\|sCAG1__21 | complete | 7563 | bp |
| gnl\|KIT\|sCAG1__27 | complete | 5122 | bp |
| gnl\|KIT\|sCAG1__29 | complete | 4718 | bp |
| gnl\|KIT\|sCAG1__31 | complete | 3547 | bp |
| gnl\|KIT\|sCAG1__32 | complete | 3497 | bp |
| gnl\|KIT\|sCAG1__33 | complete | 3289 | bp |
| gnl\|KIT\|sCAG1__36 | complete | 2950 | bp |
| gnl\|KIT\|sCAG1__37 | complete | 2755 | bp |
| gnl\|KIT\|sCAG1__38 | complete | 2744 | bp |
| gnl\|KIT\|sCAG1__49 | complete | 1563 | bp |
| gnl\|KIT\|sCAG1__53 | complete | 1399 | bp |
| gnl\|KIT\|sCAG1__57 | complete | 1228 | bp |
| gnl\|KIT\|sCAG1__58 | complete | 1216 | bp |
| gnl\|KIT\|sCAG1__59 | complete | 1114 | bp |
| gnl\|KIT\|sCAG1__61 | complete | 1071 | bp |
| gnl\|KIT\|sCAG1__62 | complete | 1047 | bp |
| gnl\|KIT\|sCAG1__65 | complete | 1007 | bp |
| gnl\|KIT\|sCAG1__68 | complete | 937 | bp |
| gnl\|KIT\|sCAG1__71 | complete | 910 | bp |
| gnl\|KIT\|sCAG1__73 | complete | 875 | bp |
| gnl\|KIT\|sCAG1__74 | complete | 864 | bp |
| gnl\|KIT\|sCAG1__78 | complete | 794 | bp |
| gnl\|KIT\|sCAG1__79 | complete | 769 | bp |
| gnl\|KIT\|sCAG1__80 | complete | 763 | bp |
| gnl\|KIT\|sCAG1__82 | complete | 759 | bp |
| gnl\|KIT\|sCAG1__83 | complete | 756 | bp |
| gnl\|KIT\|sCAG1__84 | complete | 750 | bp |
| gnl\|KIT\|sCAG1__85 | complete | 743 | bp |
| gnl\|KIT\|sCAG1__89 | complete | 680 | bp |
| gnl\|KIT\|sCAG1__91 | complete | 663 | bp |
| gnl\|KIT\|sCAG1__92 | complete | 659 | bp |
| gnl\|KIT\|sCAG1__95 | complete | 628 | bp |
| gnl\|KIT\|sCAG1__98 | complete | 622 | bp |
| gnl\|KIT\|sCAG1__100 | complete | 606 | bp |
| gnl\|KIT\|sCAG1__102 | complete | 567 | bp |
| gnl\|KIT\|sCAG1__105 | complete | 529 | bp |
| gnl\|KIT\|sCAG1__106 | complete | 519 | bp |
| gnl\|KIT\|sCAG1__108 | complete | 509 | bp |
| gnl\|KIT\|sCAG1__109 | complete | 503 | bp |
| gnl\|KIT\|sCAG1__111 | complete | 491 | bp |
| gnl\|KIT\|sCAG1__113 | complete | 486 | bp |
| gnl\|KIT\|sCAG1__114 | complete | 480 | bp |

| | | |
|---|---|---|
| gnl\|KIT\|sCAG1__115 | complete | 480 bp |
| gnl\|KIT\|sCAG1__117 | complete | 468 bp |
| gnl\|KIT\|sCAG1__119 | complete | 458 bp |
| gnl\|KIT\|sCAG1__122 | complete | 441 bp |
| gnl\|KIT\|sCAG1__123 | complete | 437 bp |
| gnl\|KIT\|sCAG1__127 | complete | 421 bp |
| gnl\|KIT\|sCAG1__128 | complete | 420 bp |
| gnl\|KIT\|sCAG1__132 | complete | 393 bp |
| gnl\|KIT\|sCAG1__133 | complete | 388 bp |
| gnl\|KIT\|sCAG1__135 | complete | 362 bp |
| gnl\|KIT\|sCAG1__136 | complete | 361 bp |
| gnl\|KIT\|sCAG1__137 | complete | 361 bp |
| gnl\|KIT\|sCAG1__138 | complete | 358 bp |
| gnl\|KIT\|sCAG1__139 | complete | 355 bp |
| gnl\|KIT\|sCAG1__140 | complete | 358 bp |
| gnl\|KIT\|sCAG1__142 | complete | 337 bp |
| gnl\|KIT\|sCAG1__144 | complete | 334 bp |
| gnl\|KIT\|sCAG1__145 | complete | 312 bp |
| gnl\|KIT\|sCAG1__147 | complete | 327 bp |
| gnl\|KIT\|sCAG1__148 | complete | 326 bp |
| gnl\|KIT\|sCAG1__149 | complete | 326 bp |
| gnl\|KIT\|sCAG1__150 | complete | 327 bp |
| gnl\|KIT\|sCAG1__152 | complete | 321 bp |
| gnl\|KIT\|sCAG1__153 | complete | 321 bp |
| gnl\|KIT\|sCAG1__154 | complete | 316 bp |
| gnl\|KIT\|sCAG1__155 | complete | 316 bp |
| gnl\|KIT\|sCAG1__156 | complete | 312 bp |
| gnl\|KIT\|sCAG1__158 | complete | 309 bp |
| gnl\|KIT\|sCAG1__159 | complete | 308 bp |
| gnl\|KIT\|sCAG1__161 | complete | 307 bp |
| gnl\|KIT\|sCAG1__162 | complete | 306 bp |
| gnl\|KIT\|sCAG1__163 | complete | 305 bp |
| gnl\|KIT\|sCAG1__164 | complete | 303 bp |
| gnl\|KIT\|sCAG1__165 | complete | 303 bp |
| gnl\|KIT\|sCAG1__166 | complete | 302 bp |
| gnl\|KIT\|sCAG1__167 | complete | 300 bp |
| gnl\|KIT\|sCAG1__168 | complete | 298 bp |
| gnl\|KIT\|sCAG1__169 | complete | 296 bp |
| gnl\|KIT\|sCAG1__170 | complete | 300 bp |
| gnl\|KIT\|sCAG1__171 | complete | 292 bp |
| gnl\|KIT\|sCAG1__172 | complete | 268 bp |
| gnl\|KIT\|sCAG1__173 | complete | 290 bp |
| gnl\|KIT\|sCAG1__174 | complete | 287 bp |
| gnl\|KIT\|sCAG1__175 | complete | 286 bp |
| gnl\|KIT\|sCAG1__176 | complete | 276 bp |

| | | | |
|---|---|---|---|
| gnl\|KIT\|sCAG1__177 | complete | 283 | bp |
| gnl\|KIT\|sCAG1__178 | complete | 282 | bp |
| gnl\|KIT\|sCAG1__179 | complete | 280 | bp |
| gnl\|KIT\|sCAG1__180 | complete | 280 | bp |
| gnl\|KIT\|sCAG1__181 | complete | 280 | bp |
| gnl\|KIT\|sCAG1__182 | complete | 279 | bp |
| gnl\|KIT\|sCAG1__183 | complete | 277 | bp |
| gnl\|KIT\|sCAG1__184 | complete | 277 | bp |
| gnl\|KIT\|sCAG1__185 | complete | 275 | bp |
| gnl\|KIT\|sCAG1__186 | complete | 273 | bp |
| gnl\|KIT\|sCAG1__187 | complete | 273 | bp |
| gnl\|KIT\|sCAG1__188 | complete | 272 | bp |
| gnl\|KIT\|sCAG1__189 | complete | 271 | bp |
| gnl\|KIT\|sCAG1__190 | complete | 270 | bp |
| gnl\|KIT\|sCAG1__191 | complete | 268 | bp |
| gnl\|KIT\|sCAG1__192 | complete | 268 | bp |
| gnl\|KIT\|sCAG1__193 | complete | 267 | bp |
| gnl\|KIT\|sCAG1__194 | complete | 267 | bp |
| gnl\|KIT\|sCAG1__195 | complete | 262 | bp |
| gnl\|KIT\|sCAG1__196 | complete | 262 | bp |
| gnl\|KIT\|sCAG1__197 | complete | 261 | bp |
| gnl\|KIT\|sCAG1__198 | complete | 260 | bp |
| gnl\|KIT\|sCAG1__199 | complete | 260 | bp |
| gnl\|KIT\|sCAG1__200 | complete | 260 | bp |
| gnl\|KIT\|sCAG1__201 | complete | 259 | bp |
| gnl\|KIT\|sCAG1__202 | complete | 259 | bp |
| gnl\|KIT\|sCAG1__204 | complete | 256 | bp |
| gnl\|KIT\|sCAG1__205 | complete | 256 | bp |
| gnl\|KIT\|sCAG1__206 | complete | 256 | bp |
| gnl\|KIT\|sCAG1__207 | complete | 256 | bp |
| gnl\|KIT\|sCAG1__208 | complete | 255 | bp |
| gnl\|KIT\|sCAG1__209 | complete | 254 | bp |
| gnl\|KIT\|sCAG1__210 | complete | 253 | bp |
| gnl\|KIT\|sCAG1__211 | complete | 252 | bp |
| gnl\|KIT\|sCAG1__212 | complete | 250 | bp |
| gnl\|KIT\|sCAG1__213 | complete | 250 | bp |
| gnl\|KIT\|sCAG1__214 | complete | 249 | bp |
| gnl\|KIT\|sCAG1__215 | complete | 249 | bp |
| gnl\|KIT\|sCAG1__216 | complete | 248 | bp |
| gnl\|KIT\|sCAG1__217 | complete | 248 | bp |
| gnl\|KIT\|sCAG1__218 | complete | 247 | bp |
| gnl\|KIT\|sCAG1__219 | complete | 247 | bp |
| gnl\|KIT\|sCAG1__220 | complete | 236 | bp |
| gnl\|KIT\|sCAG1__221 | complete | 235 | bp |
| gnl\|KIT\|sCAG1__222 | complete | 245 | bp |

| | | | |
|---|---|---|---|
| gnl\|KIT\|sCAG1__223 | complete | 245 | bp |
| gnl\|KIT\|sCAG1__224 | complete | 245 | bp |
| gnl\|KIT\|sCAG1__225 | complete | 244 | bp |
| gnl\|KIT\|sCAG1__226 | complete | 244 | bp |
| gnl\|KIT\|sCAG1__227 | complete | 244 | bp |
| gnl\|KIT\|sCAG1__228 | complete | 243 | bp |
| gnl\|KIT\|sCAG1__229 | complete | 242 | bp |
| gnl\|KIT\|sCAG1__230 | complete | 240 | bp |
| gnl\|KIT\|sCAG1__231 | complete | 240 | bp |
| gnl\|KIT\|sCAG1__232 | complete | 240 | bp |
| gnl\|KIT\|sCAG1__233 | complete | 240 | bp |
| gnl\|KIT\|sCAG1__234 | complete | 239 | bp |
| gnl\|KIT\|sCAG1__235 | complete | 239 | bp |
| gnl\|KIT\|sCAG1__236 | complete | 238 | bp |
| gnl\|KIT\|sCAG1__237 | complete | 214 | bp |
| gnl\|KIT\|sCAG1__238 | complete | 221 | bp |
| gnl\|KIT\|sCAG1__239 | complete | 235 | bp |
| gnl\|KIT\|sCAG1__240 | complete | 228 | bp |
| gnl\|KIT\|sCAG1__241 | complete | 235 | bp |
| gnl\|KIT\|sCAG1__243 | complete | 234 | bp |
| gnl\|KIT\|sCAG1__244 | complete | 234 | bp |
| gnl\|KIT\|sCAG1__245 | complete | 229 | bp |
| gnl\|KIT\|sCAG1__246 | complete | 229 | bp |
| gnl\|KIT\|sCAG1__247 | complete | 228 | bp |
| gnl\|KIT\|sCAG1__248 | complete | 227 | bp |
| gnl\|KIT\|sCAG1__249 | complete | 224 | bp |
| gnl\|KIT\|sCAG1__250 | complete | 218 | bp |
| gnl\|KIT\|sCAG1__251 | complete | 217 | bp |
| gnl\|KIT\|sCAG1__252 | complete | 216 | bp |
| gnl\|KIT\|sCAG1__253 | complete | 209 | bp |
| gnl\|KIT\|sCAG1__255 | complete | 207 | bp |
| gnl\|KIT\|sCAG1__256 | complete | 202 | bp |
| gnl\|KIT\|sCAG1__258 | complete | 205 | bp |
| gnl\|KIT\|sCAG1__261 - 395 | complete | 202 | bp each (--> 27270 bp total) |
| gnl\|KIT\|sCAG1__396 - 437 | complete | 201 | bp each (--> 8442 bp total) |
| gnl\|KIT\|sCAG1__438 - 479 | complete | 200 | bp each (--> 8402 bp total) |
